# Supplementary material for: Factors promoting sustainability of NURHI programme activities in Ilorin and Kaduna, Nigeria: findings from a qualitative study among health facility staff
Source: BMJ Open. 2020 Oct 29;10(10):e034482. doi: 10.1136/bmjopen-2019-034482 (PMC7597523; doi:10.1136/bmjopen-2019-034482)
Supplement: Supplementary data [file bmjopen-2019-034482supp001.pdf]

Appendix 1

Data Analysis for the NURHI Sustainability study

Codes were developed using both deductive and inductive approaches and codebook development was informed by guidelines on "Codebook Development for Team-Based Qualitative Analysis" by McQueen et al (1998) (33). This improved inter-coder agreement and facilitated coding and data analysis by the MLE and in-country team. Code development commenced as soon as the first set of transcripts were available. The qualitative team leads read through each transcript and developed an initial list of codes and code definitions to discuss with the larger coding team. The codes were then applied to a set of transcripts and additional codes and sub-codes were developed, and the list of codes refined as needed. This process was repeated until a final list of codes and code definitions was agreed on. Examples of quotes relating to each code were provided and reviewed following which the final codebook was agreed upon and shared with the coding team. The data collection team leads and interviewers were also involved in the process of code development and provided additional clarification as required.

Coding of the transcripts was conducted by a coding team comprised of a team lead and six coders. Two coders first independently coded one transcript and this was presented and reviewed by the coding team. Discrepancies in coding were extensively discussed and the codes to be used in these instances agreed on by the team. The remaining transcripts were then assigned to members of the coding team. Each transcript was initially coded by one person following which another coder independently reviewed the coded transcript. When discrepancies were noted, the coding team discussed these and the code to be applied was agreed on. The team lead independently conducted spot checks on randomly selected transcripts during the coding process. Coding continued until appropriate codes were applied to all segments of the transcripts (29). For the current paper, the authors read the coded transcripts again and developed additional sub-codes where required. Codes were categorized into themes for each of the objectives and cross-case analysis performed using ATLAS.ti (version 7).

Appendix II

Trustworthiness of the qualitative data

To ensure credibility of our data, the qualitative study was led by qualitative experts from Nigeria and a range of data collection methods were employed to obtain comprehensive and accurate information. We selected a range of respondents in order to obtain rich and in-depth information and facilitate triangulation of responses across the respondents and the questions in the data collection guides were framed to ensure detailed information was provided. The initial findings were also discussed with members of the core team and other researchers who were knowledgeable in the FP field and this provided opportunity for peer scrutiny. During the analysis phase, the investigators referred to the field notes to obtain additional information and clarifications, conducted member checks as necessary and compared their findings to other related studies. To improve transferability of methods, the results were interpreted within the context of the study sites. Our use of a protocol that all investigators made input into promoted dependability of our findings. Furthermore, the data collection process was conducted in line with the protocol and this process can be replicated. To ensure confirmability of our findings, the investigators conducted member checks, regularly discussed the findings with one another and with the data collection staff to ensure that the ideas of the respondents were retained, and investigators did not interpret the findings based on their own pre-formed ideas. Our findings are thus comprehensive and contribute significantly to the existing body of knowledge on sustainability of donor-funded projects
