# Supplementary material for: Factors promoting sustainability of NURHI programme activities in Ilorin and Kaduna, Nigeria: findings from a qualitative study among health facility staff
Source: BMJ Open. 2020 Oct 29;10(10):e034482. doi: 10.1136/bmjopen-2019-034482 (PMC7597523; doi:10.1136/bmjopen-2019-034482)
Supplement: Author's manuscript [file bmjopen-2019-034482.draft_revisions.pdf]

BMJ Open

BMJ Open is committed to open peer review. As part of this commitment we make the peer review history of every article we publish publicly available.

When an article is published we post the peer reviewers' comments and the authors' responses online. We also post the versions of the paper that were used during peer review. These are the versions that the peer review comments apply to.

The versions of the paper that follow are the versions that were submitted during the peer review process. They are not the versions of record or the final published versions. They should not be cited or distributed as the published version of this manuscript.

BMJ Open is an open access journal and the full, final, typeset and author-corrected version of record of the manuscript is available on our site with no access controls, subscription charges or pay-per-view fees (http://bmjopen.bmj.com).

If you have any questions on BMJ Open's open peer review process please email info.bmjopen@bmj.com

BMJ Open

Sustainability of NURHI Program Activities: Qualitative Findings

Journal:	*BMJ Open*
Manuscript ID	bmjopen-2019-034482
Article Type:	Research
Date Submitted by the Author:	04-Oct-2019

Complete List of Authors:	Olumide, Adesola; University of Ibadan/ University College Hospital, Ibadan, Institute of Child Health; University of Ibadan, Center for Population and Reproductive Health, College of Medicine,<br>McGuire, Courtney; University of North Carolina at Chapel Hill, Carolina Population Center,<br>Calhoun, Lisa; University of North Carolina at Chapel Hill, Carolina Population Center,<br>Speizer, Ilene; Univ N Carolina, MCH; MEASURE Evaluation, Carolina Population Center<br>Mumuni, Tolulope; University of Ibadan, College of Medicine, Center for Population and Reproductive Health<br>Ojengbede, Oladosu; University of Ibadan and University College Hospital, Ibadan, Department of Obstetrics and Gynaecology; University of Ibadan, Center for Population and Reproductive Health, College of Medicine,

Keywords:	PUBLIC HEALTH, Family Planning, Contraceptive, Reproductive Health

SCHOLARONE™
Manuscripts

I, the Submitting Author has the right to grant and does grant on behalf of all authors of the Work (as defined in the below author licence), an exclusive licence and/or a non-exclusive licence for contributions from authors who are: i) UK Crown employees; ii) where BMJ has agreed a CC-BY licence shall apply, and/or iii) in accordance with the terms applicable for US Federal Government officers or employees acting as part of their official duties; on a worldwide, perpetual, irrevocable, royalty-free basis to BMJ Publishing Group Ltd ("BMJ") its licensees and where the relevant Journal is co-owned by BMJ to the co-owners of the Journal, to publish the Work in this journal and any other BMJ products and to exploit all rights, as set out in our licence.

The Submitting Author accepts and understands that any supply made under these terms is made by BMJ to the Submitting Author unless you are acting as an employee on behalf of your employer or a postgraduate student of an affiliated institution which is paying any applicable article publishing charge ("APC") for Open Access articles. Where the Submitting Author wishes to make the Work available on an Open Access basis (and intends to pay the relevant APC), the terms of reuse of such Open Access shall be governed by a Creative Commons licence – details of these licences and which Creative Commons licence will apply to this Work are set out in our licence referred to above.

Other than as permitted in any relevant BMJ Author's Self Archiving Policies, I confirm this Work has not been accepted for publication elsewhere, is not being considered for publication elsewhere and does not duplicate material already published. I confirm all authors consent to publication of this Work and authorise the granting of this licence.

For peer review only

Sustainability of NURHI Program Activities: Qualitative Findings*

*Adesola Olumide[1,2], Courtney McGuire[3], Lisa M. Calhoun[3], Ilene S. Speizer[3,4], Tolulope Mumuni[1], Oladosu Ojengbede[1,5]

1. *Center for Population and Reproductive Health, College of Medicine, University of Ibadan*
2. *Institute of Child Health, College of Medicine, University of Ibadan and University College Hospital, Ibadan*
3. *Carolina Population Center, University of North Carolina at Chapel Hill*
4. *Department of Maternal and Child Health, Gillings School of Global Public Health, University of North Carolina at Chapel Hill*
5. *Department of Obstetrics and Gynaecology,, University of Ibadan and University College Hospital, Ibadan*

*Corresponding author

Corresponding author's email: daisyolu@yahoo.co.uk

For peer review only

Abstract

Objectives

This paper examines the sustainability of the Nigerian Urban Reproductive Health Initiative (NURHI) intervention activities implemented in six urban sites in Nigeria from 2009 to 2014. Under a second phase of the NURHI program, (NURHI II), activities ceased operations in four of the original six sites in 2015 (Ilorin, Abuja, Benin City, and Zaria), and continued in two sites (Kaduna and Ibadan).

Methods

A qualitative study that utilized In-depth interviews were conducted with 31 staff from 15 health facilities in two of the NURHI sites: Ilorin and Kaduna.

Results

Study findings reveal that although some of the NURHI activities, such as integration of family planning into existing maternal and child health and HIV services, sustained, many activities, such as outreach and trainings, had discontinued or were no longer being implemented at the same level as during the initial project. This situation was reported more in Ilorin than in Kaduna, and often attributed to inadequate finances for implementation of activities. The majority of the sustained activities were supported by other non-governmental organizations or special government intervention projects; this is not a true reflection of one of the characteristics of sustainability which is independent functioning.

Conclusions

Demand for family planning services generated by NURHI activities were still high in both cities. A gradual close-out of donor projects with concomitant input from government and indigenous institutions could be useful in sustaining donor activities. Furthermore, interventions to prevent a complete discontinuation of NURHI activities are required to sustain short-term achievements and ensure that the long-term effects of family planning use are attained.

Article summary

Strengths and limitations of this study

- The study was led by investigators who have experience with designing and implementing qualitative studies and analyzing qualitative data. The findings reported are thus credible
- Our choice of qualitative methods provided deeper insights into factors that promote or hinder sustainability of donor-funded programs.
- The study utilized qualitative research methods and this limits over-generalization of the findings. However, a range of participants who were knowledgeable in the issues under study were interviewed thus ensuring that findings are robust and credible

For peer review only

Keywords: Family planning, Contraceptive, Reproductive Health, NURHI, Program sustainability, Urban health

Introduction

Consistent use of modern family planning methods has been found to have health, social and economic benefits for mothers, their children and society at large (1-7). Family planning (FP) benefits include reduced risk of maternal mortality and morbidity, improved infant and child health outcomes, and improved health and well-being of families (1-5). Family planning has been identified as a key intervention to meet all of the Sustainable Development Goals (6). Despite these clear benefits, family planning use still remains low in many developing countries especially in sub-Saharan Africa (1, 8).

Tsui and colleagues (2017) examined data on modern FP* use by married women ages 15 to 49 years across four time periods: 1960–1979, 1980–1994, 1995–2004 and 2005–2015, and demonstrated that modern contraceptive prevalence rates (mCPR) were consistently much lower in Africa compared to other world regions (9). In Nigeria, modern FP use in 2013 was low with 11.1% of all women aged 15 to 49 years currently using a modern contraceptive method (10). Disaggregation of the Nigerian national data revealed substantial discrepancies in rates of modern contraceptive use across socio-economic status and region. Rates were lowest among women with no formal education, in the lowest wealth quintiles, those residing in rural areas and those in the North Eastern region of the country (10).

While data frequently show a concentration of better health and development indices in urban as compared to rural areas, they also show when disaggregated that there are areas within urban centers that have less access to health and social amenities with attendant poorer health indices (11-13). In 2009, the Urban Reproductive Health Initiative (URHI), funded by the Bill & Melinda Gates Foundation, was inaugurated with the broad goal of addressing and meeting the FP needs of the urban poor in four countries (14, 15). The URHI was implemented in select urban areas in India, Kenya, Nigeria, and Senegal. In Nigeria, the first phase of the Nigerian Urban Reproductive Health Initiative (NURHI) was implemented between 2009-2014 in the Federal Capital Territory (FCT), Ibadan, Ilorin, Kaduna, Benin and Zaria. The overall goal of the NURHI was to promote innovative FP delivery approaches and to increase modern contraceptive use in the six cities, with a focus on the urban poor. In 2015, the second phase of NURHI commenced with operations in three states: Lagos, Kaduna and Oyo. NURHI-2 expanded its focus beyond the initial urban approach to include state-level interventions in both rural and urban areas.

---

* Data on contraceptive use was obtained from the UN World Contraceptive Use 2010 database which utilized data from DHS and other national surveys of the study countries. Modern contraceptive methods included the following: barrier methods (male and female condom, foam, gel), injectables, oral contraceptive pills, implants, Intra-Uterine Devices (IUDs)

For peer review only

Description of NURHI intervention

The NURHI intervention utilized a three-tiered intervention model comprising advocacy, demand generation and service delivery to achieve its goals (16). In addition, the Measurement, Learning & Evaluation (MLE) Project undertook household and facility-based surveys to evaluate the interventions in all study cities (14).

NURHI advocacy interventions: These activities engaged various categories of stakeholders –to obtain their buy-in and create a supportive environment that would drive and sustain demand and supply of FP information and services. The advocacy-focused interventions included: (i) the use of an Advocacy Core Group (ACG) to promote and conduct FP advocacy at all levels of governance and within the community; (ii) development and distribution of advocacy materials, (iii) training in advocacy skills, (iv) national and state level policy dialogues, (v) budget tracking and (vi) development and distribution of policy briefs.

Demand generation: NURHI demand generation activities, such as the "Get It Together" campaign, were designed to improve perceptions and knowledge of modern FP methods and provide information on sources of FP services within the community. Other demand-generation activities included: branding and distribution of materials and media campaigns, mobilization of urban youth and informative radio drama series.

Improvement in service delivery: This comprised the following activities:

(i) Health system strengthening such as the NURHI 72-hour makeover, community outreaches, integration of FP services within existing maternal, neonatal and child health (MNCH) and HIV services; improving the Commodities Logistics Management System (CLMS) and setting up a referral system.

(ii) Quality improvement: This was achieved through competency-based training, integrated supportive supervision and on-the-job training, site orientation for NURHI high volume sites and distance education.

(iii) Promotion of Public Private Partnerships (PPP) to improve referrals across sectors and increase access to FP services.

These interventions were implemented from 2010 to 2014 and mid- and end-line evaluations conducted revealed that the immediate outcomes were attained (17-19). However, beyond the immediate outcomes, it is important to assess the sustainability of the NURHI interventions when funding ends. This informs whether immediate gains are maintained and whether there are longer-term health and socio-economic

For peer review only

benefits of the FP program. It is also important to highlight areas that might require urgent interventions to maintain the achievements.

The NURHI Sustainability Study was therefore conducted to assess and compare the sustainability of NURHI's interventions in a city where NURHI activities came to a close in 2015 (Ilorin) and a city where activities continued as part of NURHI phase 2 (Kaduna), and also compare these with findings in a non-NURHI-intervention city (Jos). The study had two components: a quantitative component (reported elsewhere) (20, 21); and a qualitative component, the focus of this paper. Specifically, the qualitative study sought to:

i. Determine which of the NURHI-I interventions were sustained and which were not sustained.
ii. Explore the factors that promoted continuation of NURHI activities in the intervention facilities and cities.

This paper focuses on the qualitative findings from service providers in facilities included in the NURHI-phase one activities in Ilorin and Kaduna cities only.

Methods

The qualitative phase of the NURHI Sustainability Study was led by researchers at the Centre for Population and Reproductive Health, College of Medicine, University of Ibadan, Nigeria with technical support from the Measurement, Learning & Evaluation project at the Carolina Population Center in Chapel Hill, North Carolina, USA. Information was obtained from participants in Ilorin, Kwara state and Kaduna city, Kaduna state. Data from the 2015 endline cross-sectional data collected as part of the Measurement, Learning & Evaluation project's evaluation of the NURHI program demonstrated that among all women, the percentage using a modern method was 24% in the city of Ilorin and 22% in the city of Kaduna (19).

For the qualitative study, we undertook key informant interviews (KII) with government stakeholders, in-depth interviews (IDI) with health facility staff, and focus group discussions with women from the study communities. This paper focuses on the in-depth interview data from facility staff. While in the facilities, interviewers also documented the availability and state of interventions implemented during the NURHI I program using a structured checklist. These data collection methods were selected to enable investigators to obtain in-depth information from a range of stakeholders about the NURHI I interventions and underlying factors which facilitated or undermined sustainability. The methods also facilitated triangulation of findings across the different categories of interviewees.

Study Participants

For peer review only

Selection of the facilities for the qualitative enquiry was informed by data obtained from the quantitative phase and in addition, selection required that the NURHI program had worked in the facility during its first phase of implementation. In total, NURHI worked in 21 facilities in Ilorin and 23 facilities in Kaduna under NURHI 1. Quantitative data from facilities and providers which were collected in 2010, 2014 and 2017 were analyzed to ensure that a mix of facilities categorized as high and low sustaining were selected. Factors taken into consideration included variables that would have been influenced by NURHI programming such as availability of IEC materials, training of staff within the last one year, contraceptive method availability, existence of an outreach program, and existence of a quality assurance committee. Based on this process, in-depth interviews were undertaken with facility staff in ten facilities in Ilorin and six in Kaduna between June and July, 2018. A total of 31 facility staff (20 in Ilorin and 11 in Kaduna) were interviewed including the medical director and a FP provider in each of the selected facilities. The total number of participants interviewed in the study was deemed adequate to meet the study objectives, however, there was provision to recruit additional interviewees until saturation was attained (22).

Study instruments

The in-depth-interview guide and health facility checklist were developed by a consultative process and all researchers agreed on the final version. The instruments were translated into Yoruba and Hausa, the predominant indigenous languages in the study cities, and back-translated to ensure the original meanings were retained (23). A three-day training was conducted for all research staff. The training comprised didactic lectures, case studies, role plays as well as practical field visits and interviews followed by presentations and feedback. The training was interactive and provided data collectors and the study investigators the opportunity to edit the instruments where necessary and discuss city specific issues which could arise and how to resolve these. In addition, the instruments were further reviewed following the training to ensure that locally acceptable terms were incorporated as required. The interviews were conducted by a team comprising an interviewer and a notetaker. The interviews were digitally recorded and the notetaker documented notes as well as non-verbal cues.

Analysis

Data analysis was guided by a constant comparison approach (24, 25) and conducted by a team led by a qualitative researcher from MLE and an in-country researcher. Recorded interviews and discussions were translated and transcribed verbatim. Random checks of up to 10% of all transcripts against the recordings were conducted by the analysis team leads to ensure that the interviews were accurately transcribed. The transcripts were then uploaded into ATLAS.ti software (Scientific Software, Berlin; V.7) and coded.

For peer review only

Codes were developed using both deductive and inductive approaches and codebook development was informed by guidelines on “Codebook Development for Team-Based Qualitative Analysis” by McQueen et al (1998) (26). This improved inter-coder agreement and facilitated coding and data analysis by the MLE and in-country team. Code development commenced as soon as the first set of transcripts were available and the qualitative team leads from both sites read through these transcripts and developed an initial list of codes and code definitions and discussed this with the larger coding team. The codes were then applied to a set of transcripts and additional codes and sub-codes were developed, and the list of codes refined as needed. This process was repeated until a final list of codes and code definitions was agreed on. Examples of quotes relating to each code were provided and reviewed following which the final codebook was agreed upon and shared with the coding team. The data collection team leads and interviewers were also involved in the process of code development and provided additional clarification as required.

Coding of the transcripts was conducted by a coding team comprised of a team lead and six coders. Two coders first independently coded one transcript of a KII and one of an IDI and this was presented and reviewed by the coding team. Discrepancies in coding were extensively discussed and the codes to be used in these instances agreed on by the team. The remaining transcripts were then assigned to members of the coding team. Each transcript was initially coded by one person following which another coder independently reviewed the coded transcript. When discrepancies were noted, the coding team discussed these and the code to be applied was agreed on. The team lead independently conducted spot checks on randomly selected transcripts during the coding process. Coding continued until appropriate codes were applied to all segments of the transcripts (22). For the current paper, the authors read the coded transcripts again and developed additional sub-codes where required. Codes were categorized into themes for each of the objectives and cross-case analysis performed using ATLAS.ti (version 7).

Ethical approval for the study was obtained from the National Research Ethics Committee (protocol number: NHREC Protocol Number NHREC/01/01/2007-25/04/2017) and the Institutional Review Board at the University of North Carolina at Chapel Hill (study number: 17-1215). All participants provided verbal consent and they were assured that their responses were confidential and they could discontinue the interview at any point if they desired to do this. In addition, the interviews were all conducted in private locations where participants were free to express their opinions.

Patient or Public Involvement

Research assistants were recruited from the general public and participated in the data collection. Patients were not involved in the study.

Results

For peer review only

Characteristics of participants

Thirty-one participants (20 FP providers in Ilorin and 11 in Kaduna) provided consent and were interviewed. One participant who initially agreed to take part in the study later declined for personal reasons.

Sustainability of NURHI interventions

Information on intervention activities that sustained as well as factors promoting or undermining sustainability of the NURHI-I intervention activities was obtained. Participants' responses are presented under three main themes based on the major NURHI intervention arms – advocacy, demand generation and service delivery.

Sustainability of NURHI advocacy interventions:

NURHI undertook advocacy interventions at the community, state and national levels. Members of the Advocacy Core Group (ACG) were responsible for promoting support for FP support among key stakeholders at the state level as well as community gatekeepers and religious and traditional leaders. The mobilizers attached to the ACG were also involved in community level advocacy and community mobilization as part of demand generation activities. Generally, the interviewees reported that the community-level advocacy activities undertaken by members of the Advocacy Core Group were no longer being implemented as they were during NURHI I. The decline in implementation of these activities was predominately reported by participants in Ilorin, where NURHI I had closed out, although some participants in Kaduna also noted that there had been a decline in community-level advocacy activities. The decline in activities reported in Kaduna could be due to the fact that activities under NURHI II covered a broader range of facilities including rural communities beyond Kaduna city and NURHI II did not target private facilities. Hence some respondents were not feeling the effect of lower intensity of NURHI II activities as compared to under NURHI I. A major reason for the reduction in advocacy activities were lack of funds to cover transportation and stipends for the volunteer mobilizers in the Advocacy Core Group. An excerpt from the interview with a FP provider in Ilorin demonstrates these challenges:

Interviewer: … … …, "why you were not able to maintain the advocacy"?

Respondent: nobody to give them [community Advocacy Core Group members] that stipend

An encouraging finding was that respondents in both cities lauded the long-term effects of these activities. They explained that the effects of the advocacy efforts of NURHI directed at policy makers, community and religious leaders were still being felt. Some of these effects included improved awareness and support for FP by religious and other community leaders and this had led to improved demand for FP commodities, especially long acting reversible contraceptives (LARC). A FP provider in Ilorin remarked,

For peer review only

"... … before, there is not much of awareness; before [the] NURHI come to picture. People didn't know much about family planning, even people that know, they just know maybe about the pills and injectable but now that people are aware that there is long acting, they tend to go for long acting than the short ones though we have those people that have busy schedule, like those people working in the bank, you don't see them before but now you see them coming for those long acting [methods], they can come for IUCD or Implanol". - FP Provider, Ilorin

Factors sustaining advocacy activities:

Findings revealed that, although many of the advocacy activities had discontinued since the NURHI I closeout, some were still being implemented. Interviewees provided insights into factors that promoted sustainability of the advocacy interventions including support from other non-governmental organizations (NGOs) within the state. For example, NGOs such as Marie Stopes, Pathfinder International, Planned Parenthood Federation of Nigeria (PPFN) and Society for Family Health (SFH) were currently facilitating advocacy and community mobilization in selected communities. According to a FP provider in Ilorin, "Then the Pathfinder [International] is also helping because we meet them at thee, ehm ACG [Advocacy Core Group] group that I mentioned the other time. … … … At least they provide fund for them to carry out advocacy and do other necessary things".

Sustainability of demand generation interventions:

Similar to the advocacy activities, the NURHI demand generation activities were noted to have been very successful at creating demand but the activities had also declined following the conclusion of NURHI I. This finding was reported more in Ilorin than in Kaduna. Respondents in both Ilorin and Kaduna cited the "Get it together" edutainment radio programme as being very successful in improving FP knowledge and subsequently improving perceptions and demand for FP commodities. The radio programme had however been discontinued in Ilorin following closeout of NURHI I.

As mentioned earlier, community mobilization activities especially outreach activities were not taking place to the same degree as they were during the NURHI I implementation in Ilorin because of lack of funds for the mobilizers. This was illustrated by one respondent from Ilorin:

Respondent: we don't dwell [depend] on ehn…mobilization again; people are just coming in. Unlike before when they [NURHI] were on ground, the mobilizers will go around

For peer review only - http://bmjopen.bmj.com/site/about/guidelines.xhtml

the town, they invite people, they give them cards to come in to this facility but now that NURHI has left, nothing like that again

A FP provider in Ilorin described the state of things since NURHI I closeout:

"… … … you know when NURHI was here, they helped us with many things, you know they have social mobilisers, these social mobilisers, they gave them token money for them to go out, go inside the town, call people to come and do family planning. We had the ACG they gave us money to do everything; but now nobody to finance us and people if you don't finance and ask them to do something they will not do it, because you that asked them to do it, they will think you are collecting something, so! Since the time … although when they were here, let me correct myself, the population of clients then were higher. The moment they left, things fell apart. But now, when another this thing came, this Mariestopes, then we are improving because before in this clinic we have 500, 600 clients per month this same thing declined to 100+, but now we have improved to 200, 300"

A FP provider in Kaduna explained that outreach activities had been very useful in increasing demand for FP, however the outreaches were no longer being conducted as before since NURHI I ended, " … … in the outreach, mostly it is done in the facility. Which is rather in-reach to me but then, when NURHI phase one was on, they do go to community like that and they look for a strategic place, within the community and bring in people" (FP Provider Kaduna)

Factors sustaining demand generation initiatives:

Providers in Ilorin cited outreaches, community mobilization and awareness creation activities sponsored by other NGOS within the state as being important for sustaining FP demand generation. In addition, staff in the health education unit in some facilities had incorporated FP information into their routine awareness creation activities, facilitating mobilization and service demand. A FP provider in Ilorin also cited the efforts of a youth volunteer undertaking the one-year compulsory National Youth Service Corps scheme who conducted community-based FP awareness creation as part of her personal community development activities. Examples of responses from FP providers in Ilorin explaining factors promoting sustainability of demand generation activities in Ilorin are below:

Interviewer: Okay. Do you go out of the facility? Maybe around this environment?

Respondent: Yes. … we have health educators too that go out and errhhh

Interviewer: Educate people?

For peer review only

Respondent: Educate people: ... so we have educators that … give them enlightenment on family planning, immunization and so many other things.

-FP Provider, Ilorin

"… we use to do outreach, we send some people out to go and tell people, house-to-house …, just to encourage them to come out for this family planning and then we use … especially the Marie Stopes - you know Marie Stopes has taken over after the NURHI has left … … …" – FP provider, Ilorin

Observation in the facilities in Ilorin and Kaduna revealed that many of the FP Information, Education and Communication (IEC) materials provided by NURHI were still available and not yet exhausted. In addition, other NGOs had also provided IEC materials and this facilitated distribution and display of the materials in the health facilities. The "Get It Together" radio series had discontinued in Ilorin but was still being run as part of NURHI II activities in Kaduna. It is thus possible that people were still able to listen to the programme beyond the immediate vicinity of Kaduna.

Sustainability of Service Delivery Interventions:

An important NURHI I intervention undertaken to strengthen the health system was the 72-hour makeover which involved facility renovation and supply of equipment. A few respondents in both cities however mentioned that their facilities did not benefit from the makeovers. The 72-hour makeover renovations had been somewhat sustained, for instance, some of the equipment supplied as part of the 72-hour makeover were still available and functional. Examples of explanatory quotes from FP providers in Ilorin and Kaduna are below:

"The equipment is still intact and all other things that they put in place like, the scale, equipment to check their BP, hmn … The equipment they supplied". (FP provider, Ilorin)

"We are maintaining what they gave us to work with like the instrument, like the chairs outside, if it breaks we need to repair it to make everything go well … …" (FP provider, Kaduna)

None of the respondents in both Ilorin and Kaduna reported that their facilities had experienced another 72-hour makeover or any similar large-scale maintenance since the NURHI I intervention. A provider mentioned that some minor maintenance had been undertaken in the facility. A provider in Kaduna however commented that not much had changed since NURHI I ended, "we have not been seen some things again from them. Like all these chair are from NUHRI and the tables and some of the things are from NURHI in

For peer review only - http://bmjopen.bmj.com/site/about/guidelines.xhtml

fact like the chairs the table and all these things in the facility in this unit is from NURHI, but now we no longer see anything again". Another FP provider in Kaduna commented, "as you can see, it's not long they repaired this place. I actually did the repairs. From my micro plan now, I did the repairs".

As part of the study, interviewers observed the premises of the facilities and these findings largely corroborated the responses of the interviewees (Table 1). The environment in most facilities surveyed was clean and they all had FP IEC materials displayed and NURHI-IEC materials were seen in all but one facility in Kaduna.

Table 1: Findings from facility observation by interviewers

	**Ilorin**	**Kaduna**
	n = 10	n = 6
	n (%)	n (%)
NURHI-72 hour makeover conducted	6 (60%)	5 (83.3)
NURHI 72-hour makeover plaque sighted	4 (66.7)	3 (60.0)
Clean and well-kept FP environment	10(100.0)	5 (83.3)
Conducive space that affords auditory and visual privacy allocated to FP	8 (80.0)	3 (50.0)
FP IEC materials seen	10 (100.0)	6 (100.0)
FP IEC materials produced by NURHI seen	10 (100.0)	5 (83.3)
FP IEC materials targeting adolescents and youth seen	1 (10.0)	1(16.7)
FP IEC materials produced by NURHI targeting adolescents and youth seen	1 (100.0)	0 (0)

A successful service delivery intervention which appeared to have been sustained was the integration of FP into maternal, neonatal and child health (MNCH) and HIV services. This was reported to be ongoing in both Ilorin and Kaduna. Family planning providers further explained that staff in these other units were supportive of FP services, regularly provided information to their clients on FP and also referred clients for long acting FP methods.

In both cities, participants' responses on availability of commodities indicated challenges. While many remarked that stock outs had become frequent compared to the period of the NURHI I intervention when commodity stockouts were rarely experienced. The problem of stockout was more frequently reported in Ilorin than in Kaduna. Family planning providers in private and tertiary health facilities did not appear to

For peer review only

have problems with stockout of commodities. A FP provider in a tertiary facility in Ilorin explained, “At least we don’t have stock outs. We have all the methods on ground now. We don’t have stock out and they can come for any method they like. In this facility, we offer all methods”. A provider in a private facility in Kaduna also remarked, “… once it is end of the month, I prepare a report so that there wouldn’t be any stock out”.

An interesting observation was that although the respondents often said they did not experience stockouts, their responses revealed that, virtually all of them, in both cities, had experienced stockout of at least one of the methods since NURHI I came to a close and they had been constrained to offer clients an alternate method different from what the clients requested for pending the time until the preferred method was available. Some of the interviewees’ responses are outlined below:

Respondent: Since I've been here, I have not experienced any stock out, since February. Except the month that ehnm … … … “Depo” injection [Depo Provera], that injectable that is very scarce.

Interviewer: … … , so during that period when the Depo wasn't available, what did you do to clients or what do you for clients that come to seek that particular method.

Respondent: so, hmm, we still advised them [to take] Noristerat to … we told them that if “Depo” is available we [will] give them...

- FP Provider, Ilorin

Interviewer: so [how normal] do you normally experience stock out ma or you don't experience stock out ma?

Respondent: I experienced, that's what I told you. Earlier this year even at present if I show you our cupboard there is no implanol because the one that we have - it has finished so we don't have and I requested, made requisition from (my) supervisor she told me that it's not available and the IUCD too is not available.

– FP Provider, Ilorin

“The problem of stockout we normally have will be from the whole state, once in a while, but not always. There was a time implanon was not forth coming and they will say they have not collected delivery from the Federal; so it will be a general issue … and the microgynon, we used to have stockout of microgynon, … sometimes, not always”

- FP Provider, Kaduna

For peer review only

Many of the respondents had also experienced stockout of consumables such as cottonwool, gloves, needles and syringes, bleach etc. This was more frequently reported by respondents in Ilorin than Kaduna. This was a common problem reported by many FP service providers. Stock out of consumables was less common in private facilities as they billed patients for services including FP services.

Interventions to ensure quality of FP service provision such as competency-based training, integrated supportive supervision, on-the-job training and site orientation for NURHI high volume sites had been significantly affected by the NURHI close out. Facility staff in Ilorin reported that the NURHI-type of trainings had become non-existent. An additional problem was that a good number of those who had been trained had either retired, were approaching retirement or had relocated to other health facilities or states where they would be better remunerated. A provider in Ilorin stated, “At this time I want to implore the NGOs on the training and retraining of staff on this FP. There are many staff that (they) have interest on it, when there is no training for them, there is nothing we can do. Training and retraining is very, very necessary”.

Factors promoting sustainability of service delivery interventions:

A number of the health system strengthening interventions were generally sustained. This was probably because these types of interventions potentially have effects that are long lasting and thus would probably survive for some period following close out of the intervention. Another factor that promoted sustainability of the service delivery interventions was the improved capacity of the staff to prepare budgets and forecast for commodities. This enhanced capacity was described as an outcome of the trainings provided by NURHI to key FP providers. Additional factors promoting sustainability of other service delivery interventions in Ilorin and in Kaduna (in facilities that were not included in the NURHI II intervention or where providers stated that the impact of NURHI activities had waned under NURHI II) included: NGO (Pathfinder International, SFH, Marie Stopes International) support, special government-initiated interventions such as the Saving One Million Lives Initiative, ad hoc training by FP providers who had been trained by NURHI, training by other NGOs in the states and introduction of some fees for consumables. Some funds were also made available by the government for minor repairs. In addition, one of the providers in Kaduna mentioned that he and other colleagues sometimes utilized their transportation stipends to purchase minor equipment. He explained, “I was able to get this… from what they give us, our transport, some gallipots, forceps … …”

Other examples of FP providers’ efforts to sustain the training are cited below:

Interviewer: So they will have to train that person on family planning?

Respondent: That was what NURHI was doing, but NURHI is not available now, so who will do it? Except those people that are on ground will train that person.

- FP Provider, Ilorin

Interviewer: Thank you ma. How did they contribute to family planning delivery in this facility? As in … training, resources, financial resources etc. … … the Marie Stope that you mentioned, how did they contribute to family planning service delivery in this facility?

Respondent: Thank you. Through their trainings then those that they trained, trained others too, then the knowledge people gain is a sort of contribution to family planning especially on these new methods.

FP Provider, Ilorin

Factors that helped to mitigate stockout of commodities and consumables in Ilorin and in some facilities in Kaduna were support by NGOs and government funds.

Interviewer: Has the state government or other organizations assisted with minimizing stock-out in the last five years?

Respondent: Not really, it's just partners like Marie Stopes in collaboration with the state, then this NURHI, PPFN - FP Provider, Kaduna

An FP provider from Kaduna commented, "the state government are actually trying they make sure that there is no stock out". In addition, some staff had been constrained to introduce minimal fees for consumables to keep services going. An example of a response from a provider in Ilorin on how they attempt to alleviate the stock out of consumables in Ilorin is below:

Interviewer: … … … please can you describe the funding sources for family planning

Respondent: we are not getting any fund from anybody, we are taking [a] token from the client because of this ehm … consumables. Because of the Jik, the lotion we use for cleaning, the cotton wool, the syringe, I mean.. ehn… cotton wool and some other, … … … some other [of the] consumables.

Interviewer: **…** if I may ask ma, sorry ma, like how much is the token you take from the clients

Respondent: 100 naira [0.33USDollars]

Discussion

The paper describes the extent to which the activities of NURHI I have been sustained three years after close-out of the initiative in one city and in another city where the program continued. While sustainability, described as, "the extent to which evidence-based interventions can deliver it's intended benefits over an extended period of time after external support from the donor agency is terminated" (27) is the desired goal of program interventions, this does not always happen resulting in wastage of resources and the potential for community distrust in future programmes (28). Many factors key to achieving sustainability of health interventions including use of a strategic approach for the intervention, adequate funds, community ownership and mobilization and working with existing community resources (such as manpower, social institutions and sound infrastructure) and have been suggested. (29, 30) (28, 31, 32).

The study findings revealed that although some of the activities had been sustained, many more had either been discontinued or were not being conducted to the same degree reported while the project was on-going or in the immediate close-out phase of NURHI I (17, 18). This was the situation across the three core intervention domains of advocacy, demand generation and service delivery. In addition, the reduction in sustainability of activities was reported more by respondents in Ilorin compared to their counterparts in Kaduna. This was probably because NURHI activities came to a close in 2015 in Ilorin (a NURHI I site), whereas activities are ongoing in Kaduna as part of NURHI II, although there were some private facilities that did not continue under NURHI II.

On the whole, activities that had not been sustained or were inadequately sustained included community-level advocacy activities that had been reduced because of lack of funds. Demand generation activities such as the "Get it together" radio edutainment programme in Ilorin, and community mobilization activities; as well as some service delivery activities notably training of staff, regular supply of commodities and consumables and facility-led community service outreaches had also not been sustained. This is contrary to the situation in the immediate close-out period of NURHI I reported in the NURHI I end-line survey which revealed that many of these activities were still being implemented (19). Common reasons given for failure to sustain these activities were financial constraints or irregular supply, which could also in some way be linked to funds. These findings support Shediac-Rizkallah and Bone's 1998 assertion that in addition to the availability of immediate project funds, maintenance and recurrent costs, it is important for projects to have strategies to facilitate gradual financial self-sufficiency in order to promote sustainability (33). Other authors have similarly underscored the importance of funding in sustaining health and other project/programme interventions (33-37).

Engaging government and other key partners is another important factor that helps to promote sustainability of interventions (38). The NURHI I activities included a robust advocacy component that worked with the

For peer review only

government, other partners and community members. This advocacy drive was useful in facilitating the creation of several government guidelines and strategies such as the Nigeria Family Planning Blueprint (Scale-Up Plan) 2014 (39), a dedicated FP budget line and increased fund allocation to FP by the Nigerian government. With these government interventions in place, it is expected that there would have been adequate funding to continue the NURHI project activities. This was however not the case and a possible explanation might be that although these policies and guidelines had been approved, the systems to ensure prompt implementation at all levels, especially down to the health facility levels, were still nascent and yet to be fully operational. This might thus explain the fund constraints resulting in inability to maintain community mobilization activities and uninterrupted supply of commodities and consumables. Information from a 2017 factsheet on financing of FP in Nigeria emphasized that insufficient FP funds and donor dependency were still prevalent in the country (40). A more gradual close-out of NGO-driven activities to allow the government to fully takeover the program, as suggested by a stakeholder in Kaduna, might have provided more time for the mechanisms for ensuring that the effects of the approved increment in government funding for FP are felt at the facility level and this might have prevented the decline in activities observed in Ilorin. The role of community mobilization could also have been assigned to Junior Community Health Extension Workers (J-CHEWs) who are already on government payroll in some government health facilities. These J-CHEWs could then have continued the mobilization activities on closeout of NURHI.

Another activity that had not been sustained was the training of FP providers and relevant staff. This has implications for sustained quality service and the respondents also stated that they were experiencing a decline in the pool of trained staff due to retirement and resignation of staff who were transferring their services to other establishments to avail of better remuneration. The dwindling trained workforce was reported more in Ilorin than in Kaduna. Interventions to retain staff and train staff in order to maintain a critical pool of staff trained to deliver services are thus essential. Notably, where training continued, another NGO had stepped in to fill the gap. This is reassuring, however, suggests the need for a better system for sustained training of staff in the scenario where continued NGO support may not be available.

On a positive note, some of the health systems strengthening interventions such as integration of FP services within existing Maternal, Neonatal and Child Health (MNCH) and HIV services and some aspects of the 72-hour makeovers were essentially sustained both in Ilorin and in Kaduna. Interventions that strengthen health systems typically have long term benefits and can also help to sustain other activities and project outcomes (33-35). Another positive finding was that despite the decline in activities, the respondents in both Ilorin and Kaduna acknowledged demand for FP services was still sustained as a result of the NURHI advocacy and community mobilization and engagement activities. Reviews of literature on programme sustainability have also identified community engagement and participation as factors that promote

programme sustainability (32). It is important to note however that the recurrent stockout of commodities and consumables experienced in both Ilorin and to some extent in Kaduna can potentially erode these gains. It is thus important for the issue of stock out to be urgently addressed and for measures to be put in place in the NURHI-2 sites to prevent re-occurrence of these problems.

Limitations

We acknowledge the limitations inherent in qualitative research methods and thus we do not attempt to over-generalise our study findings. However, we have reported comprehensive and in-depth findings obtained through rigorous qualitative methods.

Trustworthiness of Data

The design, data collection and analysis were informed by guidelines for ensuring trustworthiness of qualitative data described by Guba (1981) (41) and Shenton (2004) (42). These guidelines outline four issues (credibility, transferability, dependability, and confirmability) as being key to achieving trustworthiness of qualitative data. To ensure credibility of our data, the qualitative study was led by qualitative experts from Nigeria and a range of data collection methods were employed to obtain comprehensive and accurate information. We selected a range of respondents in order to obtain rich and in-depth information and facilitate triangulation of responses across the respondents and the questions in the data collection guides were framed to ensure detailed information was provided. The initial findings were also discussed with members of the core team and other researchers who were knowledgeable in the FP field and this provided opportunity for peer scrutiny. During the analysis phase, the investigators referred to the field notes to obtain additional information and clarifications, conducted member checks as necessary and compared their findings to other related studies. To improve transferability of methods, the results were interpreted within the context of the study sites. Our use of a protocol that all investigators made input into promoted dependability of our findings. Furthermore, the data collection process was conducted in line with the protocol and this process can be replicated. To ensure confirmability of our findings, the investigators conducted member checks, regularly discussed the findings with one another and with the data collection staff to ensure that the ideas of the respondents were retained, and investigators did not interpret the findings based on their own pre-formed ideas. Our findings are thus comprehensive and contribute significantly to the existing body of knowledge on sustainability of donor-funded projects.

Conclusion and recommendations

On the whole, results of this sustainability study revealed that while some of the NURHI initiated interventions were sustained many more had declined or discontinued, particularly in Ilorin where the program ended. Additional observations were that although activities were not being carried out as before,

For peer review only

their effects such as improved demand for family planning commodities and improved perception about family planning were still being felt.

In view of the overall findings we proffer the following recommendations:

(i) The government needs to put systems in place to ensure commodities and consumables are provided to government facilities as required in order to prevent stockouts. This goes beyond training facility staff on commodity management, as done by NURHI, to procurement procedures that are outside the facility mandate. In addition, approved funds for FP need to be disbursed and get to the facility levels.

(ii) A gradual close-out of donor interventions with gradual take over by government could be a means of better preparing governments to take up the activities hitherto being supported by donor funding.

(iii) Beyond successfully facilitating the adoption of policy statements such as approval of a dedicated budget line and increased allocation of funding, the next step for donor agencies should be to continue advocacy efforts in order to facilitate the process of actual implementation of these policies at all levels. This is an area to focus on in future programming in order to ensure that budgetary allocation guidelines are expedited and funds are made available as due at the peripheral levels (including health facilities) where the funds are required.

(iv) Availability of funds are key to sustainability. It might thus be advisable for drivers of donor-funded interventions to work closely with the recipients to think through various options for post-project funding and come up with income-generation plans that can be introduced ahead of the end of the project life. This could better prepare the beneficiaries to sustain the project activities.

(v) For interventions that include a training component, it is beneficial to develop context relevant training manuals, strategies for a training-of-trainers approach, and consider approaches to push the training into pre-service training programs. This will help to ensure broader capacity to provide FP services following close-out of the programmes.

Funding statement

This study was funded by a grant from the Bill & Melinda Gates Foundation to the Carolina Population Center at the University of North Carolina at Chapel Hill. This research also received support from the Population Research Infrastructure Program (P2C HD050924) awarded to the Carolina Population Center at The University of North Carolina at Chapel Hill by the Eunice Kennedy Shriver National Institute of

For peer review only

Child Health and Human Development. The contents of this article are solely the responsibility of the authors and do not necessarily represent the official views of CPC or the Bill & Melinda Gates Foundation.

Competing interests statement

Authors have no known potential conflict of interest. An abstract based on our findings has been presented at the International Family Planning Conference in Rwanda

Authors' contribution

AO was the in-country study supervisor for the study and contributed to the review of the protocol, editing and revision of the study instruments, training and supervision of field staff, data collection and analysis.

CM was the qualitative project investigator and involved in development of the protocol and study instruments, training and supervision of field staff and data analysis.

LC was a project investigator and involved in development of the protocol and study instruments and data analysis

IS was the study Principal Investigator and provided oversight for the entire project. She was also involved in development of the protocol and study instruments and review of the data.

TB was the in-country field supervisor and contributed to the review of the protocol, editing and revision of the study instruments, training and supervision of field staff, data collection and review of the data.

OO was the in-country principal investigator and provided in-country oversight for the project. He contributed to the review of the protocol, editing and revision of the study instruments, training of field staff supervision of data collection and review of the data

All authors contributed to the write-up and revision of the manuscript and approved the final version submitted for publication

Acknowledgements

We acknowledge all study participants and the research staff who worked on the project

Data Availability

The dataset associated with the paper are available on request from the Project Principal Investigator.

References

1. Ahmed S, Li Q, Liu L, Tsui AO. Maternal deaths averted by contraceptive use: an analysis of 172 countries. The Lancet. 2012;380(9837):111-25.

For peer review only

2. Canning D, Schultz TP. The economic consequences of reproductive health and family planning. The Lancet. 2012;380(9837):165-71.
3. Cates W, Karim QA, El-Sadr W, Haffner DW, Kalema-Zikusoka G, Rogo K, et al. Family planning and the millennium development goals. Science. 2010;329(5999):1603-.
4. Cleland J, Bernstein S, Ezeh A, Faundes A, Glasier A, Innis J. Family planning: the unfinished agenda. The Lancet. 2006 2006/11/18/;368(9549):1810-27.
5. Cleland J, Conde-Agudelo A, Peterson H, Ross J, Tsui A. Contraception and health. The Lancet. 2012;380(9837):149-56.
6. Starbird E, Norton M, Marcus R. Investing in family planning: key to achieving the sustainable development goals. Global Health: Science and Practice. 2016;4(2):191-210.
7. Creanga AA, Gillespie D, Karklins S, Tsui AO. Low use of contraception among poor women in Africa: an equity issue. Bulletin of the World Health Organization. 2011;89:258-66.
8. Cleland JG, Ndugwa RP, Zulu EM. Family planning in sub-Saharan Africa: progress or stagnation? Bulletin of the World Health Organization. 2011;89:137-43.
9. Tsui AO, Brown W, Li Q. Contraceptive Practice in Sub-Saharan Africa. Population and development review. 2017;43(Suppl Suppl 1):166-91. PubMed PMID: 29081552. Epub 03/31.
10. National Population Commission (NPC) [Nigeria] and ICF International. Nigeria Demographic and Health Survey 2013. Abuja, Nigeria and Rockville, Maryland, USA: NPC and ICF International, 2014.
11. Mercado S, Havemann K, Sami M, Ueda H. Urban poverty: an urgent public health issue. Journal of urban health : bulletin of the New York Academy of Medicine. 2007;84(3 Suppl):i7-i15. PubMed PMID: 17464567. Epub 04/27.
12. Elsey H, Manandah S, Sah D, Khanal S, MacGuire F, King R, et al. Public Health Risks in Urban Slums: Findings of the Qualitative 'Healthy Kitchens Healthy Cities' Study in Kathmandu, Nepal. PloS one. 2016;11(9):e0163798-e. PubMed PMID: 27685999.
13. Shetty P. Health care for urban poor falls through the gap. The Lancet. 2011;377(9766):627-8.
14. NURHI Nigerian Urban Reproductive Health Initiative. NURHI Overview 2013 [cited 2019 4 February, 2019].
15. Guilkey D, Speizer I, Lance P. Study Design for the Measurement, Learning & Evaluation Project. Project for the Urban Reproductive Health Initiative, ed. 2009.
16. Krenn S, Cobb L, Babalola S, Odeku M, Kusemiju B. Using behavior change communication to lead a comprehensive family planning program: the Nigerian Urban Reproductive Health Initiative. Global health, science and practice. 2014;2(4):427-43. PubMed PMID: 25611477. eng.
17. Measurement Learning Evaluation Project Nigeria Team. Evaluation of the Nigerian Urban Reproductive Health Initiative (NURHI) Program. Studies in family planning. 2017;48(3):253-68.
18. Measurement Learning and Evaluation (MLE) Project NPCN, Data Research and Mapping Consult Ltd,. Measurement, Learning & Evaluation of the Urban Reproductive Health

Initiative: Nigeria, 2012 Midterm Survey [TWP 2-2013]. Chapel Hill, NC, USA, 2013.
19. Measurement Learning and Evaluation (MLE) Project NPCN, Data Research and Mapping Consult, Ltd,. Measurement Learning and Evaluation of the Urban Reproductive Health Initiative: Nigeria 2015 Endline Survey. [TWP 2-2015]. Chapel Hill, NC, USA: Measurement, Learning and Evaluation Project; 2015, 2015.
20. Measurement Learning & Evaluation (MLE) Project Team. Assessing Sustainability of NURHI Programming: Results from Facility-Level Survey. Chapel Hill (NC): MLE Project: November, 2018.
21. Measurement LEMPT. Assessing Sustainability of NURHI Programming: Results from Household-Level Survey. Chapel Hill (NC): MLE Project: November, 2018.
22. Hennink M, Hutter I, Bailey A. Qualitative Research Methods. London: SAGE Publications Ltd; 2011. 304 p.
23. Brislin RW. The wording and translation of research instruments. 1986.
24. Glaser B, Strauss A. The Discovery of Grounded Theory: Strategies for Qualitative Research, Chicago: Aldin Pub. Co; 1967.

For peer review only

25. Leech NL, Onwuegbuzie AJ. Qualitative data analysis: A compendium of techniques and a framework for selection for school psychology research and beyond. School Psychology Quarterly. 2008;23(4):587.
26. MacQueen KM, McLellan E, Kay K, Milstein B. Codebook Development for Team-Based Qualitative Analysis. CAM Journal. 1998;10(2):31-6.
27. Bamberger M, Cheema S. Case studies of project sustainability: implications for policy and operations from Asian experience: The World Bank; 1990.
28. Shediac-Rizkallah MC, Bone LR. Planning for the sustainability of community-based health programs: conceptual frameworks and future directions for research, practice and policy. Health education research. 1998;13(1):87-108.
29. Chambers DA, Glasgow RE, Stange KC. The dynamic sustainability framework: addressing the paradox of sustainment amid ongoing change. Implementation Science. 2013 October 02;8(1):117.
30. Banach M, Gregory PJ. Essential tasks, skills, and decisions for developing sustainable community-based programs for children, youth, and families at risk. Journal of Extension. 2001;39(5):n5.
31. Riggs K. Strategies for Sustainability of Grant-funded Programs. 2012.
32. Iwelunmor J, Blackstone S, Veira D, Nwaozuru U, Airhihenbuwa C, Munodawafa D, et al. Toward the sustainability of health interventions implemented in sub-Saharan Africa: a systematic review and conceptual framework. Implementation Science. 2016 March 23;11(1):43.
33. Shediac-Rizkallah MC, Bone LR. Planning for the sustainability of community-based health programs: conceptual frameworks and future directions for research, practice and policy. Health Educ Res. 1998;13.
34. Iwelunmor J, Newsome V, Airhihenbuwa CO. Framing the impact of culture on health: a systematic review of the PEN-3 cultural model and its application in public health research and interventions. Ethnicity Health. 2014;19.
35. Scheirer MA. Is sustainability possible? A review and commentary on empirical studies of program sustainability. American Journal of Evaluation. 2005;26(3):320-47.
36. U.S. Department of Health and Human Services Office of Adolescent Health. Building Sustainable Programs: The Framework. United States: 2014.
37. Savaya R, Spiro SE. Predictors of sustainability of social programs. American Journal of Evaluation. 2012;33(1):26-43.
38. Sarriot EG. Qualitative research to make practical sense of sustainability in primary health care projects implemented by non-governmental organizations. Int J Health Plann Manag. 2004;19.
39. Federal Government of Nigeria. Nigeria Family Planning Blueprint (Scale-Up Plan) October 2014. Abuja, Nigeria2014.
40. Health Policy Plus (HP+) Project Nigeria. Financing for FAmily Planning in Nigeria Abuja, NigeriaJune 2017.
41. Guba EG. Criteria for assessing the trustworthiness of naturalistic inquiries. Educational Technology Research and Development. 1981;29(2):75-91.
42. Shenton AK. Strategies for ensuring trustworthiness in qualitative research projects. Education for Information 2004;22:63–75.

For peer review only

Table 1 Consolidated criteria for reporting qualitative studies (COREQ): 32-item checklist

Domain 1: Research team and reflexivity	
Personal Characteristics	
1. Interviewer/facilitator Which author/s conducted the interview or focus group?	None of the authors conducted the interviews. Authors conducted the qualitative training and trained field assistants with experience in moderating and conducting indepth interviews and focus group discussions conducted the interviews
2. Credentials What were the researcher's credentials? E.g. PhD, MD	Researchers' credentials have been entered in the author section of the journal. All researchers have a postgraduate degree including Masters, PhD, Medical fellowship
3. Occupation What was their occupation at the time of the study?	Researchers and University faculty, lecturers and honorary hospital consultants, consultants, data officers
4. Gender Was the researcher male or female?	The team comprised male and female researchers
5. Experience and training What experience or training did the researcher have?	All researchers have training and experience in research conceptualization, design, implementation, monitoring and evaluation. The qualitative aspect of the study was led by researchers with experience in qualitative research methods.
Relationship with participants	
6. Relationship established Was a relationship established prior to study commencement?	A professional relationship was established. The interviewees worked in health facilities under the NURHI programme and NURHI staff at the headquarters assisted in identifying the facilities. The in-country research team also paid advocacy visits to relevant officers within the study states and sites prior to commencement of data collection to ensure community buy-in and willingness to participate in the study.
7. Participant knowledge of the interviewer	
What did the participants know about the researcher? e.g. personal goals, reasons for doing the research	The reason for the study was explained to all participants when they were contacted. This was further explained to then when they were taken through the informed consent process. Personal information about the researchers was not shared with the interviewees
8. Interviewer characteristics What characteristics were reported about the interviewer/facilitator? e.g. Bias, assumptions, reasons and interests in the research topic	Nil
Domain 2: study design	
Theoretical framework	

For peer review only

9. Methodological orientation and Theory	
What methodological orientation was stated to underpin the study? e.g. grounded theory, discourse analysis, ethnography, phenomenology, content analysis	Content analysis
Participant selection	
10. Sampling How were participants selected? e.g. purposive, convenience, consecutive, snowball	Purposive sampling was undertaken. This was the preferred sampling technique as interviewees were expected to have been aware of the programme being evaluated and also meet certain criteria necessary for them to provide informed responses for the evaluaton
11. Method of approach How were participants approached? e.g. face-to-face, telephone, mail, email	They were approached face-to-face
12. Sample size How many participants were in the study?	Data on 31 participants were analyzed for the current study
13. Non-participation How many people refused to participate or dropped out? Reasons?	Only one participant dropped out. He had a busy schedule and could not continue with the interview as his attention was requiree
Setting	
14. Setting of data collection Where was the data collected? e.g. home, clinic, workplace	Data was collected at a venue convenient for the participant. This was the office in all cases
15. Presence of non-participants Was anyone else present besides the participants and researchers?	No. For some of the senior cadre staff interviewed, other staff occasionally came in to their office to see them, however these interruptions were minimal and confidentiality of responses was ensured.
16. Description of sample What are the important characteristics of the sample? e.g. demographic data, date	Some non-identifying demographic characteristics – gender, position/ job description, state where data was collected.
Data collection	
17. Interview guide Were questions, prompts, guides provided by the authors? Was it pilot tested?	An interview guide was utilized. The guides were pretested prior to the interviews
18. Repeat interviews Were repeat interviews carried out? If yes, how many?	No
19. Audio/visual recording Did the research use audio or visual recording to collect the data?	Research used audio recording
20. Field notes Were field notes made during and/or after the interview or focus group?	Field notes were made during the interview and expanded after the interview
21. Duration What was the duration of the interviews or focus group?	Approximately an hour – about 45 minutes to more than an hour (an hour thirty minutes
22. Data saturation Was data saturation discussed?	Yes. This was discussed
23. Transcripts returned Were transcripts returned to participants for comment and/or correction?	Transcripts were not returned to participants to correct. Participants were called to clarify issues during the analysis phase and given a brief summary of their responses

For peer review only

Domain 3: analysis and findings	
Data analysis	
24. Number of data coders How many data coders coded the data?	At least two coders coded each transcript. There were a total of six coders for the entire study. The data presented in the current paper is based on KII findings only. The process of code development was informed by guidelines I the book by McQueen et al (1998) "Codebook Development for Team-Based Qualitative Analysis"
25. Description of the coding tree Did authors provide a description of the coding tree?	Not in the publication
26. Derivation of themes Were themes identified in advance or derived from the data?	Themes were derived in advance and also from the data (both deductive and inductive theme derivation was conducted)
27. Software What software, if applicable, was used to manage the data?	ATLAS.ti software (Scientific Software, Berlin; V.7)
28. Participant checking Did participants provide feedback on the findings?	Yes. Some participants were called to clarify responses and interpretations given to responses during the analysis process
Reporting	
29. Quotations presented Were participant quotations presented to illustrate the themes / findings? Was each quotation identified? e.g. participant number	Yes
30. Data and findings consistent Was there consistency between the data presented and the findings?	Yes
31. Clarity of major themes Were major themes clearly presented in the findings?	Yes
32. Clarity of minor themes Is there a description of diverse cases or discussion of minor themes?	Yes

For peer review only

BMJ Open

Factors promoting sustainability of NURHI program activities in Ilorin and Kaduna, Nigeria: Findings from a qualitative study among health facility staff

Journal:	*BMJ Open*
Manuscript ID	bmjopen-2019-034482.R1
Article Type:	Research
Date Submitted by the Author:	16-Apr-2020
Complete List of Authors:	Olumide, Adesola; University of Ibadan/ University College Hospital, Ibadan, Institute of Child Health; University of Ibadan, Center for Population and Reproductive Health, College of Medicine,<br>McGuire, Courtney; University of North Carolina at Chapel Hill, Carolina Population Center,<br>Calhoun, Lisa; University of North Carolina at Chapel Hill, Carolina Population Center,<br>Speizer, Ilene; Univ N Carolina, MCH; MEASURE Evaluation, Carolina Population Center<br>Mumuni, Tolulope; University of Ibadan, College of Medicine, Center for Population and Reproductive Health<br>Ojengbede, Oladosu; University of Ibadan and University College Hospital, Ibadan, Department of Obstetrics and Gynaecology; University of Ibadan, Center for Population and Reproductive Health, College of Medicine,
**Primary Subject Heading**:	Public health
Secondary Subject Heading:	Global health, Health policy, Public health
Keywords:	PUBLIC HEALTH, Family Planning, Contraceptive, Reproductive Health

SCHOLARONE™
Manuscripts

I, the Submitting Author has the right to grant and does grant on behalf of all authors of the Work (as defined in the below author licence), an exclusive licence and/or a non-exclusive licence for contributions from authors who are: i) UK Crown employees; ii) where BMJ has agreed a CC-BY licence shall apply, and/or iii) in accordance with the terms applicable for US Federal Government officers or employees acting as part of their official duties; on a worldwide, perpetual, irrevocable, royalty-free basis to BMJ Publishing Group Ltd ("BMJ") its licensees and where the relevant Journal is co-owned by BMJ to the co-owners of the Journal, to publish the Work in this journal and any other BMJ products and to exploit all rights, as set out in our licence.

The Submitting Author accepts and understands that any supply made under these terms is made by BMJ to the Submitting Author unless you are acting as an employee on behalf of your employer or a postgraduate student of an affiliated institution which is paying any applicable article publishing charge ("APC") for Open Access articles. Where the Submitting Author wishes to make the Work available on an Open Access basis (and intends to pay the relevant APC), the terms of reuse of such Open Access shall be governed by a Creative Commons licence – details of these licences and which Creative Commons licence will apply to this Work are set out in our licence referred to above.

Other than as permitted in any relevant BMJ Author's Self Archiving Policies, I confirm this Work has not been accepted for publication elsewhere, is not being considered for publication elsewhere and does not duplicate material already published. I confirm all authors consent to publication of this Work and authorise the granting of this licence.

For peer review only

Factors promoting sustainability of NURHI program activities in Ilorin and Kaduna, Nigeria: Findings from a qualitative study among health facility staff

*Adesola Olumide[1,2], Courtney McGuire[3], Lisa M. Calhoun[3], Ilene S. Speizer[3,4], Tolulope Mumuni[1], Oladosu Ojengbede[1,5]

1. *Center for Population and Reproductive Health, College of Medicine, University of Ibadan*
2. *Institute of Child Health, College of Medicine, University of Ibadan and University College Hospital, Ibadan*
3. *Carolina Population Center, University of North Carolina at Chapel Hill*
4. *Department of Maternal and Child Health, Gillings School of Global Public Health, University of North Carolina at Chapel Hill*
5. *Department of Obstetrics and Gynaecology,, University of Ibadan and University College Hospital, Ibadan*

*Corresponding author

Corresponding author's email: daisyolu@yahoo.co.uk

For peer review only

Abstract

Objectives

This paper examines the sustainability of the Nigerian Urban Reproductive Health Initiative (NURHI) intervention activities implemented in six urban sites in Nigeria from 2009 to early-2015. Under a second phase of the NURHI program, (NURHI-2), activities ceased operations in four of the original six sites in 2015 (Ilorin, Abuja, Benin City, and Zaria), and continued in two sites (Kaduna and Ibadan).

Methods

A qualitative study that utilized in-depth interviews was conducted with 31 service providers from 15 health facilities in two of the NURHI sites: Ilorin and Kaduna. These sites were selected to represent a city where the program continued (Kaduna) and a city where the program ended (Ilorin) to examine the sustainability of program activities two-years post program implementation.

Results

Study findings reveal that some NURHI activities, such as integration of family planning into existing maternal and child health and HIV services, were sustained in both sites. Many activities, such as outreach and trainings, had discontinued or were no longer being implemented at the same level as during initial project activities. This situation was reported more in Ilorin than in Kaduna and often attributed to inadequate financial support for family planning activities. Most of the sustained activities were supported by other non-governmental organizations or special government intervention projects; this is not a true reflection of one of the characteristics of sustainability which is independent functioning.

Conclusions

Demand for family planning services generated by NURHI activities was still high in both cities. A gradual close-out of donor projects with concomitant input from government and indigenous institutions could be useful in sustaining donor activities. Furthermore, interventions to prevent a complete discontinuation of NURHI activities are required to sustain short-term achievements and ensure that the long-term effects of family planning use are attained.

Article summary

Strengths and limitations of this study

- The study was led by investigators who have experience with designing and implementing qualitative studies and analyzing qualitative data. The findings reported are thus credible.
- Our choice of qualitative methods provided deeper insights into factors that promote or hinder sustainability of donor-funded programs.

For peer review only - http://bmjopen.bmj.com/site/about/guidelines.xhtml

- The study utilized qualitative research methods, which limits generalization of the findings. However, a range of participants who were knowledgeable in the issues under study were interviewed thus ensuring that findings are robust and credible.

- Participants from the NURHI-2 sites could have experienced some difficulty in differentiating between NURHI-1 and NURHI2 interventions. In order to minimize the likelihood of incorrect reporting, we clarified this difference to participants in the NURHI-2 site and emphasize that the questions were based on NURHI-1 interventions.

Keywords: Family planning, Contraception, Reproductive Health, Program sustainability, Urban health, Nigeria

For peer review only

Introduction

Consistent use of modern family planning (FP) methods has been found to have health, social and economic benefits for mothers, children and society at large (1-7). FP benefits include reduced risk of maternal mortality and morbidity, improved infant and child health outcomes, and improved health and well-being of families (1-5). FP has been identified as a key intervention towards meeting virtually all of the Sustainable Development Goals (6). Despite these clear benefits, FP use remains low in many developing countries especially in sub-Saharan Africa (1, 8).

Tsui and colleagues (2017) examined data on modern* contraceptive use by married women ages 15 to 49 across four time periods and demonstrated that modern contraceptive prevalence rates (mCPR) were consistently lower in Africa compared to other world regions (9). In Nigeria, data from the 2013 Nigeria Demographic and Health Survey showed that 11.1% of all women aged 15 to 49 were currently using a modern contraceptive method (10).

Sustainability and factors influencing sustainability

Several initiatives and programmes have been implemented to improve uptake of FP in developing countries (11). Many of these programmes were donor-funded and often resulted in varied successes in terms of increased FP attitudes, knowledge and uptake. The concept of sustainability, especially with respect to public health interventions, has received a lot of attention. Various authors have defined what constitutes sustainability of health interventions and further described the factors that promote sustainability. B Bamberger and Cheema (1990) defined, sustainability of health interventions as, "the extent to which an evidence-based intervention can deliver its intended benefits over an extended period of time after external support from the donor agency is terminated" (12). Abrams et al (1998) described it simply as, "whether or not something continues to work over time" (13). Shediac-Rizkallah and Bone (1998) described three measures of sustainability: (i) continued health benefits for individuals after the initial program funding ends, (ii) continuation of program activities within an organization, and (iii) continued capacity of a community to develop and deliver health promotion programs (14). A fourth measure of sustainability which refers to efforts undertaken to maintain the ideas, beliefs, principles, or values underlying a particular programme has also been described by Weiss and colleagues (15).

Schediac and Bone (1998) describe a framework which highlights three important categories for sustainability: (i) project-related factors (i.e. factors relating to the design and implementation of the project

* Modern contraceptive methods included the following: barrier methods (male and female condom, foam, gel), injectables, oral contraceptive pills, implants, Intra-Uterine Devices (IUDs)

For peer review only - http://bmjopen.bmj.com/site/about/guidelines.xhtml

such as the resources available to the project and the project activities); (ii) factors within the organization implementing the project; (iii) and factors within the broader community environment. Shelton and colleagues (2018), describe an Integrated Sustainability Framework which consists of multilevel factors that promote sustainability across multiple settings. These factors include (i) outer contextual factors, (ii) inner contextual or organizational factors, (iii) processes within the organization, (iv) characteristics of the intervention, and (v) characteristics of the project implementer (16). Other factors that have been identified as essential for achieving sustainability include the use of a strategic approach for the intervention, adequate funds, community ownership and mobilization and working with existing community resources (such as manpower, social institutions and sound infrastructure) (14, 17-20). In an attempt to ensure sustainability of interventions, projects often incorporate a combination of these factors into their intervention package. This study examines these different components of program sustainability with a focus on the sustainability of family planning programming service delivery after a funder leaves.

Description of the NURHI intervention

The Urban Reproductive Health Initiative (URHI), funded by the Bill & Melinda Gates Foundation, was inaugurated in 2009, with the broad goal of addressing and meeting the FP needs of the urban poor in four countries: India, Kenya, Nigeria, and Senegal (21, 22). In Nigeria, the first phase of the Nigerian Urban Reproductive Health Initiative (NURHI) was implemented between 2009 and early-2015 in the Federal Capital Territory (FCT), Ibadan, Ilorin, Kaduna, Benin and Zaria. The overall goal of the NURHI was to promote innovative FP delivery approaches and to increase modern contraceptive use in the six cities, with a focus on the urban poor. In 2015, the second phase of NURHI commenced with operations in three states: Lagos, Kaduna and Oyo. NURHI-2 expanded its focus beyond the initial urban approach to include state-level interventions in both rural and urban areas. NURHI-2 incorporated approaches to support sustainability of project activities and ultimately FP uptake in its intervention sites.

The NURHI intervention utilized a three-tiered intervention model comprising advocacy, demand generation and service delivery to achieve its goals (23). The focus of this paper is on the service delivery components of the NURHI program. The NURHI service delivery approach included the following activities:

(i) Health system strengthening such as the NURHI 72-hour makeover, community outreaches, integration of FP services within existing maternal, neonatal and child health (MNCH) and HIV services; improving the Commodities Logistics Management System (CLMS) and setting up a referral system.

For peer review only - http://bmjopen.bmj.com/site/about/guidelines.xhtml

(ii) Quality improvement: This was achieved through competency-based training, integrated supportive supervision and on-the-job training, site orientation for NURHI high volume sites and distance education.

(iii) Promotion of Public Private Partnerships (PPP) to improve referrals across sectors and increase access to FP services.

The Measurement, Learning & Evaluation (MLE) Project undertook the impact evaluation of the NURHI program; this included baseline, midterm, and endline household and facility-based surveys (24-26). Post immediate impact effects, it is important to assess the sustainability of the NURHI interventions when funding ends. This informs whether immediate gains are maintained and whether there are longer-term system-level and program-level benefits of the FP program. It is also important to highlight areas that might require urgent interventions to maintain the achievements.

The focus of this paper is to describe the extent to which NURHI interventions were sustained and factors influencing sustainability in a city where NURHI activities came to a close in 2015 (Ilorin) and a city where activities continued as part of NURHI-2 (Kaduna). Two measures of sustainability: (i) the extent to which the project activities continued in the target health facilities following closeout and (ii) continuation of immediate effects of the intervention on the target population after project close out

Research questions

(i) How did NURHI intervention activities change after the project was closed?

(ii) Were there changes in attitudes and demand for FP after the project closed?

Methods

The NURHI Sustainability Study had two components: a quantitative component (27, 28); and a qualitative component from which data for the current paper is obtained. The qualitative phase of the NURHI Sustainability Study was led by researchers at the Centre for Population and Reproductive Health, College of Medicine, University of Ibadan, Nigeria with technical support from the MLE project at the Carolina Population Center in Chapel Hill, North Carolina, USA. Information was obtained from participants in Ilorin, Kwara state and Kaduna city, Kaduna state.

For the qualitative study, we undertook key informant interviews (KII) with government stakeholders, in-depth interviews (IDI) with health facility staff, and focus group discussions with women from the study communities. This paper is based on the in-depth interview data from facility staff as the objective is to

For peer review only - http://bmjopen.bmj.com/site/about/guidelines.xhtml

assess sustainability of facility-based interventions. In addition to the interviews, research staff also documented the availability and state of interventions implemented during the NURHI-1 program using a structured checklist. These data collection methods were selected to enable investigators to obtain in-depth information from a range of facility stakeholders about the NURHI-1 interventions and underlying factors which facilitated or undermined sustainability. The methods also facilitated triangulation of findings across the different categories of interviewees.

Study Participants

Selection of the facilities for the qualitative enquiry was informed by data obtained from the quantitative phase. In total, NURHI worked in 21 facilities in Ilorin and 23 facilities in Kaduna under NURHI-1. Quantitative data from facilities collected in 2017 were analyzed to ensure that a mix of facilities categorized as high and low sustaining were selected. Factors taken into consideration included variables that would have been influenced by NURHI programming such as availability of IEC materials, training of staff within the last year, contraceptive method availability, existence of an outreach program, and existence of a quality assurance committee. Based on this process, in-depth interviews were undertaken with facility staff in ten facilities in Ilorin (five that had positive indication of sustainability and five with worse indicators related to sustainability) and six in Kaduna (three with better and three with worse indicators) between June and July, 2018. A total of 31 facility staff (20 in Ilorin and 11 in Kaduna) were interviewed including the medical director and a FP provider in each of the selected facilities. Only staff who had been working in the facility prior to commencement of the NURHI-1 intervention were eligible to participate. The total number of participants interviewed was deemed adequate to meet the study objectives, however, there was provision to recruit additional interviewees until saturation was attained (29).

Study instruments

The in-depth-interview guide and health facility checklist were developed by a consultative process and all researchers agreed on the final version. The instruments were translated into Yoruba and Hausa, the predominant indigenous languages in the study cities, and back-translated to ensure the original meanings were retained (30). The instruments were pretested among a comparable group of respondents and revised as required subsequently. A three-day training was conducted for all research staff who conducted the interviews. The training comprised didactic lectures, case studies, role plays as well as practical field visits and interviews followed by presentations and feedback. The training was interactive and provided data collectors and the study investigators the opportunity to edit the instruments where necessary and discuss city specific issues which could arise and how to resolve these. In addition, the instruments were further reviewed following the training to ensure that locally acceptable terms were incorporated as required. The

For peer review only - http://bmjopen.bmj.com/site/about/guidelines.xhtml

interviews were conducted by a team comprising an interviewer and a notetaker and each interview lasted approximately 45 minutes to more than an hour. The interviews were digitally recorded and the notetaker documented notes as well as non-verbal cues.

Analysis

Data analysis was guided by a constant comparison approach (31, 32) and conducted by a team led by a qualitative researcher from MLE and an in-country researcher. Recorded interviews and discussions were translated and transcribed verbatim. Random checks of up to 10% of all transcripts against the recordings were conducted by the analysis team leads to ensure that the interviews were accurately transcribed. The transcripts were then uploaded into ATLAS.ti software (Scientific Software, Berlin; V.7) and coded.

Codes were developed using both deductive and inductive approaches and codebook development was informed by guidelines on "Codebook Development for Team-Based Qualitative Analysis" by McQueen et al (1998) (33). This improved inter-coder agreement and facilitated coding and data analysis by the MLE and in-country team. The data collection team leads and interviewers were also involved in the process of code development and provided additional clarification as required.

Coding of the transcripts was conducted by a coding team comprised of a team lead and six coders. When discrepancies were noted, the coding team discussed these and the code to be applied was agreed on. The team lead independently conducted spot checks on randomly selected transcripts during the coding process. Coding continued until appropriate codes were applied to all segments of the transcripts (29). For the current paper, the authors read the coded transcripts again and developed additional sub-codes where required. Codes were categorized into themes for each of the objectives and cross-case analysis performed using ATLAS.ti (version 7); (Additional details on data analysis in Appendix I).

Ethical approval for the study was obtained from the National Research Ethics Committee (protocol number: NHREC Protocol Number NHREC/01/01/2007-25/04/2017) and the Institutional Review Board at the University of North Carolina at Chapel Hill (study number: 17-1215). All participants provided verbal consent and they were assured that their responses were confidential and they could discontinue the interview at any point if they desired to do this. In addition, the interviews were all conducted in private locations where participants were free to express their opinions.

Patient or Public Involvement

Research assistants were recruited from the general public and participated in the data collection. Patients were not involved in the study.

For peer review only - http://bmjopen.bmj.com/site/about/guidelines.xhtml

Results

Participant characteristics

Thirty-one participants (20 FP providers in Ilorin and 11 in Kaduna) provided consent and were interviewed. One participant who initially agreed to take part in the study later declined for personal reasons.

Presentation of respondents' views is organized based on the key themes and presented in the following sub-sections:

i. Sustainability of NURHI-1 activities
ii. Sustainability of outcomes that support facility use
iii. Factors promoting sustainability of the NURHI-1 intervention

Sustainability of NURHI-1 activities

In both cities, responses revealed that many of the NURHI-1 activities had either discontinued or were no longer being carried out to the same extent as occurred under NURHI-1.

<u>Sustainability of community mobilization activities</u>

Generally, the interviewees reported that the community-level advocacy activities and community mobilization undertaken by members of the Advocacy Core Group (ACG) were no longer being implemented as they were during NURHI-1. The decline in implementation of these activities was predominately reported by participants in Ilorin, where NURHI-1 had closed out, although some participants in Kaduna also noted that there had been a decline in community-level advocacy and mobilization.

An FP provider in Ilorin described the state of things since the NURHI-1 closeout:

> "…you know when NURHI was here, they helped us with many things. They had social mobilisers. These social mobilisers, they gave them token money for them to go out, to go inside the town, call people to come and do family planning. We had the ACG [Advocacy Core Group] - they gave us money to do everything; but now, [there is] nobody to finance us."

Another FP provider in Ilorin remarked,

> "We don't depend on ehn…mobilization again; people are just coming in [for services]. Unlike before, when they [NURHI] were on ground, the mobilizers will go around the town, they invite people, they give them cards to come in to this facility; but now that NURHI has left - nothing like that again."

For peer review only - http://bmjopen.bmj.com/site/about/guidelines.xhtml

Although most of those interviewed in Kaduna mentioned that community mobilization and outreaches were still ongoing, a few FP providers remarked that there had been a decline in these activities. For example, a FP provider in Kaduna explained, "…the outreach; mostly it is done in the facility. Which is rather 'in-reach' to me, but then, when NURHI phase 1 was on, they [community mobilizers] do go into the community and they look for a strategic place, within the community and bring in people".

The decline in activities reported in Kaduna could be attributed to the fact that activities under NURHI-2 covered a broader range of facilities including rural communities beyond Kaduna city and NURHI-2 did not target private facilities. Hence some respondents were not experiencing the effect of the lower intensity of NURHI-2 activities compared to the situation under NURHI-1.

Sustainability of service delivery interventions

One of the NURHI-1 service delivery interventions which appeared to have been sustained in Ilorin and Kaduna was the integration of FP services into existing maternal, neonatal and child health (MNCH) and HIV services within the intervention health facilities. FP providers explained that staff in other units such as the MNCH and general out-patients units were supportive of FP services, regularly provided information to their clients on FP and also referred clients to FP clinics for long acting FP methods.

A respondent in Kaduna affirmed,

"We've integrated it [Family planning] with the lab and [services for] people living with HIV. There is a training we undergo. That's why the PMTCT unit is just here and the FP [unit] is here. So that it will not be far, so any person coming for PMTCT, if she's a married woman, they will now advise her to come to the FP unit, so she'll come for the FP and we will counsel her. And there are some women that come here purposely for FP, then we send them to the lab for HIV test".

In both cities, responses on availability of FP commodities in the post-NURHI-1 period indicated challenges. Respondents remarked that commodity stockout had become more frequent compared to the period of the NURHI-1 intervention. This was reported more often among respondents in Ilorin than in Kaduna. For instance, a provider in Ilorin stated,

> "Earlier this year, even at present, if I show you our cupboard, there is no Implanol because the one that we have has finished, so we don't have. And I requested, I made requisition from my supervisor. She told me that it's not available and the IUCD too is not available".

For peer review only - http://bmjopen.bmj.com/site/about/guidelines.xhtml

In Kaduna, a FP provider corroborated the issue of stockout of commodities and efforts made to ameliorate this at the facility level which included reaching out to other facilities, "The facility cannot do anything. We use to go to Kaduna south sometimes their supply comes from the federal level so we can get what we want exactly. Sometimes when we request for hundred we get fifty so that is why we get the stock out."

An interesting observation was that although some respondents initially said they did not experience commodity stockout, after probing for details of availability of the different FP commodities, it was discovered that virtually all government facilities, in both Ilorin and Kaduna, had experienced stockout of at least one of the methods since NURHI-1 came to a close. Thus, providers had sometimes been constrained to offer clients an alternate method, if the preferred method was unavailable. Excerpts from interviews with FP providers illustrating this are outlined below:

Respondent: Since I've been here, I have not experienced any stock out, since February. Except the month that ehnm … … … "Depo" injection [Depo Provera], that injectable was very scarce.

Interviewer: … …, so during that period when the Depo wasn't available, what did you do for clients that came to seek that particular method?

Respondent: so, hmm, we still advised them [to take] Noristerat … … … we told them that if "Depo" is available we will give them...

- FP Provider, Ilorin

A provider in Kaduna described the situation thus:

> "The problem of stockout we normally have will be from the whole state, once in a while, but not always. There was a time Implanon was not forthcoming and they will say they have not collected delivery from the Federal [level]. So, it will be a general issue. … And the microgynon, we used to have stockout of microgynon, - sometimes, not always."

FP providers in private and tertiary health facilities in both cities did not appear to have problems with stockout of commodities. A FP provider in a tertiary facility in Ilorin explained, "…we don't have stock outs. We have all the methods on ground now. We don't have stock out and they [clients] can come for any method they like. In this facility, we offer all methods".

A provider in a private facility in Kaduna also remarked, "… once it is end of the month, I prepare a report so that there wouldn't be any stock out".

For peer review only - http://bmjopen.bmj.com/site/about/guidelines.xhtml

The problem of stock out of FP consumables was quite common both in Ilorin and Kaduna. Many of the respondents had experienced stockout of consumables such as cottonwool, gloves, needles and syringes, and bleach. A FP provider in Kaduna specifically mentioned the challenges with availability of consumables experienced since NURHI-1 transited to NURHI-2.

> "You know now, they don't concentrate more in the urban, they are now in the rural areas, so like now the consumables they used to give us has reduce. [It's] not like before, not like NURHI-1 and that is understood because they are facing the rural areas, the grassroots."

Stock out of consumables was less common in private facilities as these facilities billed patients for services including FP services. They could thus afford to procure consumables privately and not depend on government supply or supply by NGOs.

Sustainability of quality FP service provision

Interventions to ensure quality of FP service provision such as competency-based training, integrated supportive supervision, on-the-job training and site orientation for NURHI high volume sites had been significantly affected by the NURHI-1 close out. Facility staff in Ilorin reported that the NURHI-type of trainings had become non-existent. This observation was also made by FP providers in Kaduna.

A provider in Ilorin stated,

> "At this time I want to implore the NGOs on the training and retraining of staff on this FP. There are many staff that have interest, when there is no training for them, there is nothing we can do. Training and retraining are very, very necessary".

A FP provider in Kaduna also noted.

> "NURHI have not been training our staff again, that's one of the changes."

An additional problem mentioned was that a good number of the facility staff who had been trained had either retired, were approaching retirement or had relocated to other health facilities or states where they would be better remunerated.

Another important NURHI-1 intervention undertaken to strengthen the health system was the 72-hour makeover. This involved facility renovation and supply of equipment. The 72-hour makeover renovations had been somewhat sustained, for instance, some of the equipment supplied were still available and functional. Examples of quotes from FP providers in Ilorin and Kaduna are below:

For peer review only - http://bmjopen.bmj.com/site/about/guidelines.xhtml

> "The equipment is still intact and all other things that they put in place like; the scale, equipment to check their BP, hmn … The equipment they supplied" (FP provider, Ilorin)

> "We are maintaining what they gave us to work with; like the instruments, the chairs outside. If they break, we need to repair them to make everything go well." (FP provider, Kaduna)

A provider in Kaduna, mentioned that some minor maintenance which he spearheaded had been undertaken in his facility. He commented, "as you can see, it's not long they repaired this place. I actually did the repairs. From my micro plan, I did the repairs".

None of the respondents in Ilorin and in Kaduna reported that their facilities had experienced a 72-hour makeover or any similar large-scale renovation since the end of NURHI-1. A provider in Kaduna commented, "we have not been seeing some things again from them [NURHI]. Like all these chairs are from NURHI and the tables and some of the things are from NURHI. In fact like the chairs, the tables and all these things in the facility - in this unit are from NURHI, but now we no longer see anything again".

As part of the study, interviewers observed the premises of the facilities for evidence of sustainability of the 72-hour health facility renovations. These findings largely corroborated the reports of the interviewees. A higher proportion of respondents in Kaduna than in Ilorin affirmed that their facilities had benefited from the NURHI 72-hour makeover, however, the make-over plaques were seen in a higher proportion of facilities in Ilorin than in Kaduna. The environments around all the health facilities surveyed in Ilorin and most of facilities in Kaduna were clean and well-kept at the time of the visit. Many facilities in Ilorin and Kaduna had spaces allocated to FP which were conducive and offered privacy for clients. All the facilities in Ilorin and in Kaduna had FP IEC materials displayed and NURHI-IEC materials were seen in all but one facility in Kaduna (Table 1).

For peer review only - http://bmjopen.bmj.com/site/about/guidelines.xhtml

Table 1: Sustainability of facility renovations as observed by interviewers

	Ilorin	Kaduna
	n = 10	n = 6
	n (%)	n (%)
NURHI-72 hour makeover conducted	6 (60%)	5 (83.3)
NURHI 72-hour makeover plaque sighted	4 (66.7)	3 (60.0)
Clean and well-kept FP environment	10(100.0)	5 (83.3)
Conducive space that affords auditory and visual privacy allocated to FP	8 (80.0)	3 (50.0)
FP IEC materials seen	10 (100.0)	6 (100.0)
FP IEC materials produced by NURHI seen	10 (100.0)	5 (83.3)
FP IEC materials targeting adolescents and youth seen	1 (10.0)	1(16.7)
FP IEC materials produced by NURHI targeting adolescents and youth seen	1 (100.0)	0 (0)

Sustainability of outcomes that support facility use

Respondents in both cities affirmed that the NURHI-1 intervention had improved FP awareness, perceptions and demand for commodities. They further acknowledged that although NURHI activities had declined, the effects of these activities such as advocacy directed at policy makers, community and religious leaders were still being felt. An FP provider in Ilorin remarked,

> ".Before, there was not much of awareness - before NURHI came. People didn't know much about family planning. Even people that knew, they just knew maybe about the pills and injectables. But now that people are aware that there is long acting [contraceptive], they tend to go for the long acting rather than the short [acting] ones."

She further added, "We have those people that have busy schedules, like those people working in the bank, you don't see them before, but now you see them coming for those long acting [methods], they come for IUCD or Implanol".

A provider in Ilorin affirmed that demand for FP services had increased as a result of the NURHI-1 intervention.

For peer review only - http://bmjopen.bmj.com/site/about/guidelines.xhtml

"It has increased because there is more awareness now, people get to know more about family planning and during the antenatal clinic, they are getting information there. Even some, before they deliver, you will see them coming for counseling that immediately they deliver or as soon as they deliver, they want to come for family planning. So they are more knowledgeable now and this makes them to be coming." - Family planning provider, Ilorin

Similar views were expressed in Kaduna, with perspectives that demand for FP, especially LARCs, had increased, as a result of community awareness outreaches.

"More people asking for it, you know it's a long term method and people are thinking for them to go, comeback after 2 months, still go and come back after 3 months, they just feel the long acting method is the best, they just tell you I prefer the long acting method because once I just put it on, I'll just go on my own, I'll forget I'm putting it no need of me telling myself I have to go back in 2 – 3 months, so it's preferable I go for the long term method." - Family planning provider, Kaduna

The NURHI "Get It Together" edutainment radio programme was also noted to have contributed to improved awareness about FP and subsequently demand for FP commodities. This finding was reported by respondents in both Ilorin and Kaduna although the radio programme had been discontinued in Ilorin following closeout of NURHI-1.

Factors promoting sustainability of the NURHI I activities

Respondents' views about factors that helped to sustain some of the activities following close-out are presented below

Role of funding in sustainability

Virtually all respondents echoed the role of funding in promoting sustainability and the importance of funds cut across all categories of factors promoting sustainability was the. In addition to funding, we examine other factors that influenced sustainability.

Project design and implementation factors

NURHI's focus on health system strengthening activities such as training of key facility staff in FP commodity service delivery was a key factor that promoted sustainability. This improved the capacity of the staff to provide a wider range of FP commodities and subsequently improved demand for FP, especially for LARCs.

A FP provider in Ilorin explained,

"… before you can only access long acting method like implant in either teaching hospital and other places. But now, because of the training, our nurses are now having, people are now coming

For peer review only - http://bmjopen.bmj.com/site/about/guidelines.xhtml

here and we introduced that method to them. They now have access to long term [contraception], unlike before that they do not have access. Some may say they don't want intrauterine, IUCD but some go for implant so that's why it has increased."

Availability of IEC materials also contributed to sustainability of demand for FP. Observation in the facilities in Ilorin and Kaduna revealed that many of the FP IEC materials provided by NURHI were still available. These materials were also used by staff to create awareness of FP during health education sessions and outreaches.

Factors within the organizational setting

A major contributor to sustainability of some activities was the integration of NURHI interventions such as health education and awareness raising and FP services into routine clinics already being run in the different health facilities.

A family planning provider in Ilorin reported:

> "like, we normally have our National Programe on Immunization (NPI) sessions - Mondays and Wednesdays; then they talk to them on family planning during that period. Mondays, Wednesdays and Fridays. Especially for mothers who had just been, who have just given birth and they come for their routine immunization for their children. So we talk to them on family planning in this facility."

For example, staff in the health education unit in some facilities were said to have incorporated FP information into their routine awareness creation activities, facilitating continuation of some mobilization activities and ultimately FP service demand. As explained by a FP provider in Ilorin, "… … we have [health] educators that … give them enlightenment on family planning, immunization and so many other things".

Facilities in Kaduna also involved Voluntary Community Mobilizers (VCM) and Traditional Birth Attendants (TBAs) in creating awareness of FP within the community.

> "Yes, the VCM too are playing their own role, the TBA's too they are playing their own role in family planning, educating women out there at the community level, then during routine immunization where we gather many of the women that bring their children for immunization so

For peer review only - http://bmjopen.bmj.com/site/about/guidelines.xhtml

we use that opportunity too because at that time we can really get some them. Then during antenatal again, toward choosing a method of their own choice. So as soon as they deliver, then that of interval family planning so there is great improvement." (Family planning provider, Kaduna)

Staff within the health facilities were also quite motivated and had attempted to continue the NURHI activities through various means such as using personal funds, stepping down the FP training they had received from NURHI to other staff and charging minimal fees for consumables.

A FP provider in Kaduna mentioned that he and other colleagues sometimes utilized their transportation stipends to purchase minor equipment. He explained, "I was able to get these… from what they give us, our transport, some gallipots, forceps … …"

Other examples of FP providers' efforts to sustain the training are cited below:

Interviewer: So they will have to train that person on family planning?

Respondent: That was what NURHI was doing, but NURHI is not available now, so who will do it? Except those people that are on ground will train that person.

- FP Provider, Ilorin

Some staff had been constrained to introduce minimal fees for consumables to keep services going. An example of a response from a provider in Ilorin on how they attempt to alleviate the stock out of consumables in Ilorin is below:

Interviewer: Please can you describe the funding sources for family planning.

Respondent: We are not getting any fund from anybody, we are taking [a] token from the client because of this ehm … consumables. Because of the Jik, the lotion we use for cleaning, the cotton wool, the syringe, I mean.. ehn… cotton wool and some other, … … … some other [of the] consumables.

Interviewer: If I may ask ma, sorry ma, like how much is the token you take from the clients?

Respondent: 100 naira [0.33USDollars]

Factors within the larger community

These factors included availability of funds, trained manpower, commodities and materials provided by other NGO-led projects. Government support was also mentioned by some of the respondents. In addition,

For peer review only - http://bmjopen.bmj.com/site/about/guidelines.xhtml

the awareness generated by the NURHI advocacy and community mobilization programmes further played a role in sustaining demand for FP commodities.

Respondents cited other NGOs working to improve FP in their states as instrumental towards promoting sustainability of NURHI interventions in Ilorin and in Kaduna (in facilities that were not included in the NURHI-2 intervention or where providers stated that the impact of NURHI activities had waned under NURHI-2). Some of these NGOs included Pathfinder International, Society for Family Health (SFH) and Marie Stopes International. A special government-initiated intervention - Saving One Million Lives Initiative - was also cited as contributing towards sustainability of some NURHI activities in Ilorin and Kaduna. Providers in Ilorin cited outreaches, community mobilization and awareness creation activities sponsored by other NGOS within the state as being important for sustaining FP demand generation.

A FP provider in Ilorin explained,

> **"**We used to organize outreaches, we send some people out to go and tell people, house-to-house …, just to encourage them to come out for this family planning and then we use … especially the Marie Stopes - you know Marie Stopes has taken over after the NURHI has left. "

Another FP provider in Ilorin expressed concern about the initial decline in clientele experienced when NURHI-1 came to an end. She however mentioned that the number of clients seen had improved when another NGO commenced a FP intervention project in the city. She explained thus,

> "Although when they [NURHI] were here, let me correct myself, the population of clients then were higher. The moment they [NURHI] left, things fell apart. But now, when another NGO came, this Marie Stopes, then we are improving because before in this clinic we had 500, 600 clients per month this declined to 100- plus, but now we have improved to 200, 300."

Additional ways through which other NGOS had contributed to the continuation of some of the NURHI activities were through training of health care workers in the provision of FP services and provision of IEC materials. NGOs had also been instrumental towards mitigating stockout of commodities and consumables in Ilorin and in some facilities in Kaduna.

Interviewer: The Marie Stopes that you mentioned, how did they contribute to family planning service delivery in this facility?

Respondent: Thank you. Through their trainings, then those that they trained, trained others too, then the knowledge people gained was a sort of contribution to family planning

For peer review only - http://bmjopen.bmj.com/site/about/guidelines.xhtml

especially on these new methods.

FP Provider, Ilorin

Responses on government efforts to sustain the NURHI activities and provide funding for FP commodities and consumables and for minor repairs within the health facilities differed. While some respondents acknowledged government efforts, others were of the opinion that the government had not done enough but that NGOs were the main supporters of FP services. The differing opinions were expressed by respondents in both Ilorin and Kaduna.

A provider in Kaduna commented: “the state government are actually trying. They make sure that there is no stock out”. Another FP provider in Kaduna however held a contrary view and when asked if the state government or other organization had assisted with minimizing stock-out in the last five years, he responded, “not really, it’s just partners like Marie Stopes in collaboration with the state, then this NURHI, Planned Parenthood Federation of Nigeria (PPFN)”.

Discussion

The paper describes the extent to which the activities of NURHI-1 have been sustained three years after close-out of the initiative in Ilorin and in Kaduna where the program continued but with a slightly different scope and focus. Quantitative findings revealed that although some of the activities had been sustained in Ilorin, many more had either been discontinued or were not being conducted to the same degree, although contraceptive use continued to increase slightly (24, 25). Common reasons given by providers in this study for failure to sustain these activities were financial constraints or problems with the commodity supply chain, which could also be linked to funding. These findings support Shediac-Rizkallah and Bone’s 1998 assertion that in addition to the availability of immediate project funds, maintenance, and recurrent costs, it is important for projects to have strategies to facilitate gradual financial self-sufficiency in order to promote sustainability (34). Other authors have similarly underscored the importance of funding in sustaining health and other programme interventions (34-38).

Another activity that had not been sustained was the training of FP providers and relevant staff. This has implications for sustained quality of service. Respondents further stated that they were experiencing a decline in the pool of trained staff due to retirement and resignation of staff who were transferring their services to other establishments to avail of better remuneration. Interventions to retain staff and train new staff in order to maintain a critical pool of staff trained to deliver services are thus essential. Notably, where training continued, another NGO had stepped in to fill the gap. This is reassuring; however, it suggests the

For peer review only - http://bmjopen.bmj.com/site/about/guidelines.xhtml

need for a better system for sustained training of staff in the scenario where continued NGO support may not be available.

On a positive note, integration of FP services within existing Maternal, Neonatal and Child Health (MNCH) and HIV services and some aspects of the 72-hour makeover were sustained both in Ilorin and Kaduna. Interventions that strengthen health systems typically have long term benefits and can also help to sustain other activities and project outcomes (35, 36).

On the whole, challenges with sustainability of activities were reported more by respondents in Ilorin compared to their counterparts in Kaduna. This was probably because NURHI activities came to a close in 2015 in Ilorin (a NURHI-1 site), whereas activities are ongoing in Kaduna as part of NURHI-2, although there were some private facilities that did not continue under NURHI-2.

Activities most likely to have been affected by the NURHI-1 close out were those that were introduced by NURHI for example - use of Advocacy Core Group community mobilizers, family planning outreach services and the "Get It Together" media campaign. Activities that leveraged on existing structures such as training of already employed health staff, and those that were incorporated into existing services such as integrating FP service provision and health education into other routine clinics were somewhat better sustained. Integration of project activities with existing programs/services has been identified as a factor within the organizational domain that promotes sustainability(34)

Some of the immediate effects of the NURHI interventions including improvement in awareness and demand for FP appeared to have been sustained although concerns about declines in FP demand were raised by some respondents. Reviews of literature on programme sustainability have identified community engagement as a factor that promotes programme sustainability (18). It is important to note however that the recurrent stockout of commodities and consumables experienced in Ilorin and to some extent in Kaduna could potentially erode these gains. It is thus important for the issue of stock out to be urgently addressed and for measures to be put in place in the NURHI-2 sites to prevent re-occurrence of stockout.

Engaging government and other key partners is another important factor that helps to promote sustainability of interventions (39). The NURHI-1 activities included a robust advocacy component that worked with the government, other partners and community members. This advocacy drive was useful in facilitating the creation of several government guidelines and strategies such as the Nigeria Family Planning Blueprint (Scale-Up Plan) 2014 (40), a dedicated FP budget line, and increased fund allocation to FP by the Nigerian government. With these government interventions in place, it is expected that there would have been adequate funding to continue the NURHI project activities. This was however not the case and a possible explanation might be that although these policies and guidelines had been approved, the systems to ensure

For peer review only - http://bmjopen.bmj.com/site/about/guidelines.xhtml

prompt implementation at all levels, especially down to the health facility levels, were still nascent and yet to be fully operational. This might thus explain the funding constraints resulting in inability to maintain community mobilization activities and uninterrupted supply of commodities and consumables. Information from a 2017 factsheet on financing of FP in Nigeria emphasized that insufficient FP funds and donor dependency were still prevalent in the country (41). A more gradual close-out of NGO-driven activities to allow the government to fully takeover the program, as suggested by a stakeholder in Kaduna, might have provided more time for the mechanisms for ensuring that the effects of the approved increment in government funding for FP are launched and felt at the facility level; this might have prevented the decline in activities observed in Ilorin. The role of community mobilization could also have been assigned to Junior Community Health Extension Workers (J-CHEWs) who are already on government payroll in some government health facilities. These J-CHEWs could then have continued the mobilization activities on closeout of NURHI.

Limitations

We acknowledge the limitations inherent in qualitative research methods and thus we do not attempt to generalise our study findings. Study participants provided their views, and these could have been limited based on their job description and cadre within the health system. We have however reported comprehensive and in-depth findings obtained through rigorous qualitative methods and these provide useful insight into the extent to which the NURHI-1 activities were sustained.

Trustworthiness of Data

The design, data collection and analysis were informed by guidelines for ensuring trustworthiness of qualitative data described by Guba (1981) (42) and Shenton (2004) (43). These guidelines mention four issues (credibility, transferability, dependability, and confirmability) as key to achieving trustworthiness of qualitative data. The investigators made efforts to ensure the study design, data collection and analysis met these criteria (please see details in Appendix II).

Conclusion and recommendations

On the whole, results of this sustainability study revealed that while some of the NURHI initiated interventions were sustained many more had declined or discontinued, particularly in Ilorin where the program ended. Additional observations were that although activities were not being carried out as before, their effects such as improved demand for family planning commodities and improved perception about family planning were still being felt.

For peer review only - http://bmjopen.bmj.com/site/about/guidelines.xhtml

Funding statement

This study was funded by a grant from the Bill & Melinda Gates Foundation to the Carolina Population Center at the University of North Carolina at Chapel Hill. This research also received support from the Population Research Infrastructure Program (P2C HD050924) awarded to the Carolina Population Center at The University of North Carolina at Chapel Hill by the Eunice Kennedy Shriver National Institute of Child Health and Human Development. The contents of this article are solely the responsibility of the authors and do not necessarily represent the official views of CPC or the Bill & Melinda Gates Foundation.

Competing interests statement

Authors have no known potential conflict of interest. An abstract based on our findings has been presented at the International Family Planning Conference in Rwanda

Authors' contribution

AO was the in-country study supervisor for the study and contributed to the review of the protocol, editing and revision of the study instruments, training and supervision of field staff, data collection and analysis.

CM was the qualitative project investigator and involved in development of the protocol and study instruments, training and supervision of field staff and data analysis.

LC was a project investigator and involved in development of the protocol and study instruments and data analysis

IS was the study Principal Investigator and provided oversight for the entire project. She was also involved in development of the protocol and study instruments and review of the data.

TB was the in-country field supervisor and contributed to the review of the protocol, editing and revision of the study instruments, training and supervision of field staff, data collection and review of the data.

OO was the in-country principal investigator and provided in-country oversight for the project. He contributed to the review of the protocol, editing and revision of the study instruments, training of field staff supervision of data collection and review of the data

All authors contributed to the write-up and revision of the manuscript and approved the final version submitted for publication

Acknowledgements

We acknowledge all study participants and the research staff who worked on the project

Data Availability

The dataset associated with the paper are available on request from the Project Principal Investigator.

For peer review only - http://bmjopen.bmj.com/site/about/guidelines.xhtml

References

1. Ahmed S, Li Q, Liu L, Tsui AO. Maternal deaths averted by contraceptive use: an analysis of 172 countries. The Lancet. 2012;380(9837):111-25.
2. Canning D, Schultz TP. The economic consequences of reproductive health and family planning. The Lancet. 2012;380(9837):165-71.
3. Cates W, Karim QA, El-Sadr W, Haffner DW, Kalema-Zikusoka G, Rogo K, et al. Family planning and the millennium development goals. Science. 2010;329(5999):1603-.
4. Cleland J, Bernstein S, Ezeh A, Faundes A, Glasier A, Innis J. Family planning: the unfinished agenda. The Lancet. 2006 2006/11/18/;368(9549):1810-27.
5. Cleland J, Conde-Agudelo A, Peterson H, Ross J, Tsui A. Contraception and health. The Lancet. 2012;380(9837):149-56.
6. Starbird E, Norton M, Marcus R. Investing in Family Planning: Key to Achieving the Sustainable Development Goals. Global Health: Science and Practice. 2016;4(2):191-210.
7. Creanga AA, Gillespie D, Karklins S, Tsui AO. Low use of contraception among poor women in Africa: an equity issue. Bulletin of the World Health Organization. 2011;89:258-66.
8. Cleland JG, Ndugwa RP, Zulu EM. Family planning in sub-Saharan Africa: progress or stagnation? Bulletin of the World Health Organization. 2011;89:137-43.
9. Tsui AO, Brown W, Li Q. Contraceptive Practice in Sub-Saharan Africa. Population and development review. 2017;43(Suppl Suppl 1):166-91. PubMed PMID: 29081552. Epub 03/31.
10. National Population Commission (NPC) [Nigeria] and ICF International. Nigeria Demographic and Health Survey 2013. Abuja, Nigeria and Rockville, Maryland, USA: NPC and ICF International, 2014.
11. Mwaikambo L, Speizer IS, Schurmann A, Morgan G, Fikree F. What Works in Family Planning Interventions: A Systematic Review. Studies in Family Planning. 2011;42(2):67-82.
12. Bamberger M, Cheema S. Case studies of project sustainability: implications for policy and operations from Asian experience: The World Bank; 1990.
13. Abrams L, Palmer I, Hart T. Sustainability management guidelines. Pretoria: Department of Water Affairs and Forestry. 1998.
14. Shediac-Rizkallah MC, Bone LR. Planning for the sustainability of community-based health programs: conceptual frameworks and future directions for research, practice and policy. Health education research. 1998;13(1):87-108.
15. Weiss H, Coffman J, Bohan-Baker M, editors. Evaluation's role in supporting initiative sustainability. Proceedings of the Fifth Bi-Annual Meeting of the Urban Seminar Series on Children's Health and Safety at Harvard University, Cambridge, MA, USA; 2002.
16. Shelton RC, Cooper BR, Stirman SW. The sustainability of evidence-based interventions and practices in public health and health care. Annual review of public health. 2018;39:55-76.
17. Riggs K. Strategies for Sustainability of Grant-funded Programs. 2012.
18. Iwelunmor J, Blackstone S, Veira D, Nwaozuru U, Airhihenbuwa C, Munodawafa D, et al. Toward the sustainability of health interventions implemented in sub-Saharan Africa: a systematic review and conceptual framework. Implementation Science. 2016 March 23;11(1):43.
19. Banach M, Gregory PJ. Essential tasks, skills, and decisions for developing sustainable community-based programs for children, youth, and families at risk. Journal of Extension. 2001;39(5):n5.
20. Chambers DA, Glasgow RE, Stange KC. The dynamic sustainability framework: addressing the paradox of sustainment amid ongoing change. Implementation Science. 2013 October 02;8(1):117.
21. NURHI Nigerian Urban Reproductive Health Initiative. NURHI Overview 2013 [cited 2019 4 February, 2019].
22. Guilkey D, Speizer I, Lance P. Study Design for the Measurement, Learning & Evaluation Project. Project for the Urban Reproductive Health Initiative, ed. 2009.
23. Krenn S, Cobb L, Babalola S, Odeku M, Kusemiju B. Using behavior change communication to lead a comprehensive family planning program: the Nigerian Urban Reproductive Health Initiative. Global health, science and practice. 2014;2(4):427-43. PubMed PMID: 25611477. eng.

For peer review only - http://bmjopen.bmj.com/site/about/guidelines.xhtml

24. Measurement Learning Evaluation Project Nigeria Team. Evaluation of the Nigerian Urban Reproductive Health Initiative (NURHI) Program. Studies in family planning. 2017;48(3):253-68.
25. Measurement Learning and Evaluation (MLE) Project NPCN, Data Research and Mapping Consult Ltd,. Measurement, Learning & Evaluation of the Urban Reproductive Health Initiative: Nigeria, 2012 Midterm Survey [TWP 2-2013]. Chapel Hill, NC, USA, 2013.
26. Measurement Learning and Evaluation (MLE) Project NPCN, Data Research and Mapping Consult, Ltd,. Measurement Learning and Evaluation of the Urban Reproductive Health Initiative: Nigeria 2015 Endline Survey. [TWP 2-2015]. Chapel Hill, NC, USA: Measurement, Learning and Evaluation Project; 2015, 2015.
27. Measurement Learning & Evaluation (MLE) Project Team. Assessing Sustainability of NURHI Programming: Results from Facility-Level Survey. Chapel Hill (NC): MLE Project: November, 2018.
28. Measurement LEMPT. Assessing Sustainability of NURHI Programming: Results from Household-Level Survey. Chapel Hill (NC): MLE Project: November, 2018.
29. Hennink M, Hutter I, Bailey A. Qualitative Research Methods. London: SAGE Publications Ltd; 2011. 304 p.
30. Brislin RW. The wording and translation of research instruments. 1986.
31. Glaser B, Strauss A. The Discovery of Grounded Theory: Strategies for Qualitative Research, Chicago: Aldin Pub. Co; 1967.
32. Leech NL, Onwuegbuzie AJ. Qualitative data analysis: A compendium of techniques and a framework for selection for school psychology research and beyond. School Psychology Quarterly. 2008;23(4):587.
33. MacQueen KM, McLellan E, Kay K, Milstein B. Codebook Development for Team-Based Qualitative Analysis. CAM Journal. 1998;10(2):31-6.
34. Shediac-Rizkallah MC, Bone LR. Planning for the sustainability of community-based health programs: conceptual frameworks and future directions for research, practice and policy. Health Educ Res. 1998;13(1):87-108.
35. Iwelunmor J, Newsome V, Airhihenbuwa CO. Framing the impact of culture on health: a systematic review of the PEN-3 cultural model and its application in public health research and interventions. Ethnicity Health. 2014;19.
36. Scheirer MA. Is sustainability possible? A review and commentary on empirical studies of program sustainability. American Journal of Evaluation. 2005;26(3):320-47.
37. U.S. Department of Health and Human Services Office of Adolescent Health. Building Sustainable Programs: The Framework. United States: 2014.
38. Savaya R, Spiro SE. Predictors of sustainability of social programs. American Journal of Evaluation. 2012;33(1):26-43.
39. Sarriot EG. Qualitative research to make practical sense of sustainability in primary health care projects implemented by non-governmental organizations. Int J Health Plann Manag. 2004;19.
40. Federal Government of Nigeria. Nigeria Family Planning Blueprint (Scale-Up Plan) October 2014. Abuja, Nigeria2014.
41. Health Policy Plus (HP+) Project Nigeria. Financing for FAmily Planning in Nigeria Abuja, NigeriaJune 2017.
42. Guba EG. Criteria for assessing the trustworthiness of naturalistic inquiries. Educational Technology Research and Development. 1981;29(2):75-91.
43. Shenton AK. Strategies for ensuring trustworthiness in qualitative research projects. Education for Information 2004;22:63–75.

For peer review only - http://bmjopen.bmj.com/site/about/guidelines.xhtml

Appendix 1

Data Analysis for the NURHI Sustainability study

Codes were developed using both deductive and inductive approaches and codebook development was informed by guidelines on "Codebook Development for Team-Based Qualitative Analysis" by McQueen et al (1998) (33). This improved inter-coder agreement and facilitated coding and data analysis by the MLE and in-country team. Code development commenced as soon as the first set of transcripts were available. The qualitative team leads read through each transcript and developed an initial list of codes and code definitions to discuss with the larger coding team. The codes were then applied to a set of transcripts and additional codes and sub-codes were developed, and the list of codes refined as needed. This process was repeated until a final list of codes and code definitions was agreed on. Examples of quotes relating to each code were provided and reviewed following which the final codebook was agreed upon and shared with the coding team. The data collection team leads and interviewers were also involved in the process of code development and provided additional clarification as required.

Coding of the transcripts was conducted by a coding team comprised of a team lead and six coders. Two coders first independently coded one transcript and this was presented and reviewed by the coding team. Discrepancies in coding were extensively discussed and the codes to be used in these instances agreed on by the team. The remaining transcripts were then assigned to members of the coding team. Each transcript was initially coded by one person following which another coder independently reviewed the coded transcript. When discrepancies were noted, the coding team discussed these and the code to be applied was agreed on. The team lead independently conducted spot checks on randomly selected transcripts during the coding process. Coding continued until appropriate codes were applied to all segments of the transcripts (29). For the current paper, the authors read the coded transcripts again and developed additional sub-codes where required. Codes were categorized into themes for each of the objectives and cross-case analysis performed using ATLAS.ti (version 7).

For peer review only

Appendix II

Trustworthiness of the qualitative data

To ensure credibility of our data, the qualitative study was led by qualitative experts from Nigeria and a range of data collection methods were employed to obtain comprehensive and accurate information. We selected a range of respondents in order to obtain rich and in-depth information and facilitate triangulation of responses across the respondents and the questions in the data collection guides were framed to ensure detailed information was provided. The initial findings were also discussed with members of the core team and other researchers who were knowledgeable in the FP field and this provided opportunity for peer scrutiny. During the analysis phase, the investigators referred to the field notes to obtain additional information and clarifications, conducted member checks as necessary and compared their findings to other related studies. To improve transferability of methods, the results were interpreted within the context of the study sites. Our use of a protocol that all investigators made input into promoted dependability of our findings. Furthermore, the data collection process was conducted in line with the protocol and this process can be replicated. To ensure confirmability of our findings, the investigators conducted member checks, regularly discussed the findings with one another and with the data collection staff to ensure that the ideas of the respondents were retained, and investigators did not interpret the findings based on their own pre-formed ideas. Our findings are thus comprehensive and contribute significantly to the existing body of knowledge on sustainability of donor-funded projects

For peer review only

Table 1 Consolidated criteria for reporting qualitative studies (COREQ): 32-item checklist

Domain 1: Research team and reflexivity		Page numbers
Personal Characteristics		
1. Interviewer/facilitator Which author/s conducted the interview or focus group?	None of the authors conducted the interviews. Authors conducted the qualitative training and trained field assistants with experience in moderating and conducting in depth interviews and focus group discussions conducted the interviews	Page 8<br><br>“”The interviews were conducted by a team comprising an interviewer and a notetaker”<br><br>Penultimate sentence under the sub-heading, “study instruments”
2. Credentials What were the researcher’s credentials? E.g. PhD, MD	Researchers’ credentials have been entered in the author section of the journal. All researchers have a postgraduate degree including Masters, PhD, Medical fellowship	These were filled out in the online form
3. Occupation What was their occupation at the time of the study?	Researchers and University faculty, lecturers and honorary hospital consultants, consultants, data officers	This information is not in the manuscript
4. Gender Was the researcher male or female?	The team comprised male and female researchers	Information is not in the manuscript
5. Experience and training What experience or training did the researcher have?	All researchers have training and experience in research conceptualization, design, implementation, monitoring and evaluation. The qualitative aspect of the study was led by researchers with experience in qualitative research methods.	This is not in the manuscript
Relationship with participants		None
6. Relationship established Was a relationship established prior to study commencement?	A professional relationship was established. The interviewees worked in health facilities under the NURHI programme and NURHI staff at the headquarters assisted in identifying the facilities. The in-country research team also paid advocacy visits to relevant officers within the study states and sites prior to commencement of data collection to ensure community	Information is not in the paper

For peer review only - http://bmjopen.bmj.com/site/about/guidelines.xhtml

	buy-in and willingness to participate in the study.	
7. Participant knowledge of the interviewer		
What did the participants know about the researcher? e.g. personal goals, reasons for doing the research	The reason for the study was explained to all participants when they were contacted. This was further explained to then when they were taken through the informed consent process. Personal information about the researchers was not shared with the interviewees	Page 9. First paragraph on ethics
8. Interviewer characteristics What characteristics were reported about the interviewer/facilitator? e.g. Bias, assumptions, reasons and interests in the research topic	Nil. Trained research assistants conducted the interviews	
Domain 2: study design		
Theoretical framework		
9. Methodological orientation and Theory		
What methodological orientation was stated to underpin the study? e.g. grounded theory, discourse analysis, ethnography, phenomenology, content analysis	Analysis was conducted using constant comparison	Pages 4 and 8
Participant selection		
10. Sampling How were participants selected? e.g. purposive, convenience, consecutive, snowball	Purposive sampling was undertaken. This was the preferred sampling technique as interviewees were expected to have been aware of the programme being evaluated and also meet certain criteria necessary for them to provide informed responses for the evaluation	Methods Page 7 under study participants
11. Method of approach How were participants approached? e.g. face-to-face, telephone, mail, email	They were approached face-to-face	Methods Page 7 under study participants
12. Sample size How many participants were in the study?	Data on 31 participants were analyzed for the current study	Methods Page 7 under study participants
13. Non-participation How many people refused to participate or dropped out? Reasons?	Only one participant dropped out. He had a busy schedule and could not continue with the interview as his attention was requiree	Methods Page 9 under participant characteristics

For peer review only

Setting		
14. Setting of data collection Where was the data collected? e.g. home, clinic, workplace	Data was collected at a venue convenient for the participant. This was the office in all cases	Methods<br>Under study participants, page 7
15. Presence of non-participants Was anyone else present besides the participants and researchers?	No. For some of the senior cadre staff interviewed, other staff occasionally came in to their office to see them, however these interruptions were minimal and confidentiality of responses was ensured.	
16. Description of sample What are the important characteristics of the sample? e.g. demographic data, date	Some non-identifying demographic characteristics – gender, position/ job description, state where data was collected.	Methods<br>Their job description is mentioned<br>under study participants, page 7
Data collection		
17. Interview guide Were questions, prompts, guides provided by the authors? Was it pilot tested?	An interview guide was utilized. The guides were pretested prior to the interviews	Methods-Study instruments, page 7
18. Repeat interviews Were repeat interviews carried out? If yes, how many?	No	
19. Audio/visual recording Did the research use audio or visual recording to collect the data?	Research used audio recording	Methods – Page 8. Last sentence under study instruments
20. Field notes Were field notes made during and/or after the interview or focus group?	Field notes were made during the interview and expanded after the interview	Methods – Page 8. Last sentence under study instruments
21. Duration What was the duration of the interviews or focus group?	Approximately an hour – about 45 minutes to more than an hour (an hour thirty minutes	Methods – Page 8. Last sentence under study instruments
22. Data saturation Was data saturation discussed?	Yes. This was discussed	Methods-Study instruments, page 7
23. Transcripts returned Were transcripts returned to participants for comment and/or correction?	Transcripts were not returned to participants to correct. Participants were called to clarify issues during the analysis phase and given a brief summary of their responses	Trustworthiness<br>Page 22
Domain 3: analysis and findings		
Data analysis		

For peer review only

24. Number of data coders How many data coders coded the data?	At least two coders coded each transcript. There were a total of six coders for the entire study. The data presented in the current paper is based on KII findings only. The process of code development was informed by guidelines I the book by McQueen et al (1998) "Codebook Development for Team-Based Qualitative Analysis"	Methods –Analysis: page 8
25. Description of the coding tree Did authors provide a description of the coding tree?	Not in the publication	
26. Derivation of themes Were themes identified in advance or derived from the data?	Themes were derived in advance and also from the data (both deductive and inductive theme derivation was conducted)	Methods<br>- Data analysis, pages 8 and 9
27. Software What software, if applicable, was used to manage the data?	ATLAS.ti software (Scientific Software, Berlin; V.7)	Methods<br>Data analysis, page 9
28. Participant checking Did participants provide feedback on the findings?	Yes. Some participants were called to clarify responses and interpretations given to responses during the analysis process	Trustworthiness<br>Page 22
Reporting		
29. Quotations presented Were participant quotations presented to illustrate the themes / findings? Was each quotation identified? e.g. participant number	Yes	Results Pages 9 to 20
30. Data and findings consistent Was there consistency between the data presented and the findings?	Yes	Results Pages 9 to 20
31. Clarity of major themes Were major themes clearly presented in the findings?	Yes	Results Pages 9 to 20
32. Clarity of minor themes Is there a description of diverse cases or discussion of minor themes?	Yes	Results Pages 9 to 20

For peer review only

BMJ Open

Factors promoting sustainability of NURHI program activities in Ilorin and Kaduna, Nigeria: Findings from a qualitative study among health facility staff

Journal:	*BMJ Open*
Manuscript ID	bmjopen-2019-034482.R2
Article Type:	Research
Date Submitted by the Author:	12-Sep-2020
Complete List of Authors:	Olumide, Adesola; University of Ibadan College of Medicine, Institute of Child Health; University of Ibadan College of Medicine, Center for Population and Reproductive Health<br>McGuire, Courtney; University of North Carolina at Chapel Hill, Carolina Population Center,<br>Calhoun, Lisa; University of North Carolina at Chapel Hill, Carolina Population Center,<br>Speizer, Ilene; Univ N Carolina, MCH; MEASURE Evaluation, Carolina Population Center<br>Mumuni, Tolulope; University of Ibadan College of Medicine, Center for Population and Reproductive Health, College of Medicine,<br>Ojengbede, Oladosu; University of Ibadan College of Medicine, Department of Obstetrics and Gynaecology; University of Ibadan, Center for Population and Reproductive Health, College of Medicine,
**Primary Subject Heading**:	Public health
Secondary Subject Heading:	Global health, Health policy, Public health
Keywords:	PUBLIC HEALTH, Family Planning, Contraceptive, Reproductive Health

SCHOLARONE™
Manuscripts

I, the Submitting Author has the right to grant and does grant on behalf of all authors of the Work (as defined in the below author licence), an exclusive licence and/or a non-exclusive licence for contributions from authors who are: i) UK Crown employees; ii) where BMJ has agreed a CC-BY licence shall apply, and/or iii) in accordance with the terms applicable for US Federal Government officers or employees acting as part of their official duties; on a worldwide, perpetual, irrevocable, royalty-free basis to BMJ Publishing Group Ltd ("BMJ") its licensees and where the relevant Journal is co-owned by BMJ to the co-owners of the Journal, to publish the Work in this journal and any other BMJ products and to exploit all rights, as set out in our licence.

The Submitting Author accepts and understands that any supply made under these terms is made by BMJ to the Submitting Author unless you are acting as an employee on behalf of your employer or a postgraduate student of an affiliated institution which is paying any applicable article publishing charge ("APC") for Open Access articles. Where the Submitting Author wishes to make the Work available on an Open Access basis (and intends to pay the relevant APC), the terms of reuse of such Open Access shall be governed by a Creative Commons licence – details of these licences and which Creative Commons licence will apply to this Work are set out in our licence referred to above.

Other than as permitted in any relevant BMJ Author's Self Archiving Policies, I confirm this Work has not been accepted for publication elsewhere, is not being considered for publication elsewhere and does not duplicate material already published. I confirm all authors consent to publication of this Work and authorise the granting of this licence.

For peer review only

Factors promoting sustainability of NURHI program activities in Ilorin and Kaduna, Nigeria: Findings from a qualitative study among health facility staff

*Adesola Olumide[1,2], Courtney McGuire[3], Lisa M. Calhoun[3], Ilene S. Speizer[3,4], Tolulope Babawarun[1], Oladosu Ojengbede[1,5]

1. *Center for Population and Reproductive Health, College of Medicine, University of Ibadan*
2. *Institute of Child Health, College of Medicine, University of Ibadan and University College Hospital, Ibadan*
3. *Carolina Population Center, University of North Carolina at Chapel Hill*
4. *Department of Maternal and Child Health, Gillings School of Global Public Health, University of North Carolina at Chapel Hill*
5. *Department of Obstetrics and Gynaecology,, University of Ibadan and University College Hospital, Ibadan*

*Corresponding author

Corresponding author's email: daisyolu@yahoo.co.uk

For peer review only

For peer review only - http://bmjopen.bmj.com/site/about/guidelines.xhtml

Abstract

Objectives

The Nigerian Urban Reproductive Health Initiative (NURHI) was implemented in six urban sites in Nigeria from 2009 to early-2015. Under a second phase, (NURHI-2), activities ceased operations in four of the original six sites in 2015 (Ilorin, Abuja, Benin City, and Zaria), and continued in two sites (Kaduna and Ibadan). This paper examines the sustainability of facility-based intervention activities implemented under NURHI-1 in Ilorin and Kaduna.

Methods

A qualitative study that utilized in-depth interviews was conducted with 31 service providers purposively selected from 10 of the NURHI-intervention facilities in Ilorin and six in Kaduna. Interviews were digitally recorded and transcripts uploaded into ATLAS.ti for analysis. Structured observations to document renovations implemented during the NURHI-1 interventions were also conducted in the health facilities.

Results

Family planning (FP) awareness creation within the facilities and integration of FP into existing maternal and child health and HIV services, were sustained in both cities. The majority of the equipment supplied as part of the NURHI 72-hour clinic makeover were still functional in both cities. Respondents in both cities reported that FP awareness and demand were sustained. On the whole, challenges with sustaining activities were reported more among respondents in Ilorin than Kaduna. In Ilorin, NURHI outreach activities and trainings, had discontinued while in Kaduna, they were no longer being implemented to the same degree as occurred during NURHI-1. Inadequate funds was a major reason for discontinued activities in both cities while integration of FP into existing services enhanced sustainability.

Conclusions

Many activities were not sustained in Ilorin compared to Kaduna although FP awareness and demand remained high in both cities. Integration of FP into existing services promoted sustainability in Ilorin and Kaduna. A gradual close-out of donor projects with concomitant input from government and indigenous institutions could be useful in sustaining donor activities.

Article summary

Strengths and limitations of this study

- The study was led by investigators who have experience with designing and implementing qualitative studies and analyzing qualitative data. The findings reported are thus credible.

For peer review only - http://bmjopen.bmj.com/site/about/guidelines.xhtml

- Our choice of qualitative methods provided deeper insights into factors that promote or hinder sustainability of donor-funded programs.

- The study utilized qualitative research methods, which limits generalization of the findings. However, a range of participants who were knowledgeable in the issues under study were interviewed thus ensuring that findings are robust and credible.

- Participants from the NURHI-2 sites could have experienced some difficulty in differentiating between NURHI-1 and NURHI-2 interventions. To minimize the likelihood of incorrect reporting, we clarified this difference to participants in the NURHI-2 site and emphasize that the questions were based on NURHI-1 interventions.

Keywords: Family planning, Contraception, Reproductive health, Program sustainability, Urban health, Nigeria

For peer review only

For peer review only - http://bmjopen.bmj.com/site/about/guidelines.xhtml

Introduction

Consistent use of modern family planning (FP) methods has been found to have health, social and economic benefits for mothers, children and society at large (1-7). Family planning benefits include reduced risk of maternal mortality and morbidity, improved infant and child health outcomes, and improved health and well-being of families (1-5). Family planning has been identified as a key intervention towards meeting virtually all of the Sustainable Development Goals (6). Despite these clear benefits, FP use remains low in many developing countries especially in sub-Saharan Africa (1, 8).

Tsui and colleagues (2017) examined data on modern[*] contraceptive use by married women ages 15 to 49 across four time periods and demonstrated that modern contraceptive prevalence rates (mCPR) were consistently lower in Africa compared to other world regions (9). In Nigeria, data from the 2013 Nigeria Demographic and Health Survey showed that 11.1% of all women aged 15 to 49 were currently using a modern contraceptive method (10).

Sustainability and factors influencing sustainability

Several initiatives and programmes have been implemented to improve uptake of FP in developing countries (11). Many of these programmes were donor-funded and resulted in varied successes in terms of increased FP attitudes, knowledge, and uptake. The concept of sustainability, especially with respect to public health interventions, has received a lot of attention. Various authors have defined what constitutes sustainability of health interventions and further described the factors that promote sustainability. Bamberger and Cheema (1990) defined sustainability of health interventions as, “the extent to which an evidence-based intervention can deliver its intended benefits over an extended period of time after external support from the donor agency is terminated” (12). Abrams and colleagues (1998) described it simply as, “whether or not something continues to work over time” (13). Shediac-Rizkallah and Bone (1998) described three measures of sustainability: (i) continued health benefits for individuals after the initial program funding ends, (ii) continuation of program activities within an organization, and (iii) continued capacity of a community to develop and deliver health promotion programs (14). A fourth measure of sustainability which refers to efforts undertaken to maintain the ideas, beliefs, principles, or values underlying a particular programme has also been described by Weiss and colleagues (15).

Schediac and Bone (1998) developed a framework which highlights three important categories for sustainability: (i) project-related factors (i.e. factors relating to the design and implementation of the project

[*] Modern contraceptive methods included the following: barrier methods (male and female condom, foam, gel), injectables, oral contraceptive pills, implants, Intra-Uterine Devices (IUDs)

For peer review only - http://bmjopen.bmj.com/site/about/guidelines.xhtml

such as the resources available to the project and the project activities); (ii) factors within the organization implementing the project; (iii) and factors within the broader community environment. Shelton and colleagues (2018), described an Integrated Sustainability Framework which consists of multilevel factors that promote sustainability across multiple settings. These factors include (i) outer contextual factors, (ii) inner contextual or organizational factors, (iii) processes within the organization, (iv) characteristics of the intervention, and (v) characteristics of the project implementer (16). Other factors that have been identified as essential for achieving sustainability include the use of a strategic approach for the intervention, adequate funds, community ownership and mobilization and working with existing community resources (such as manpower, social institutions and sound infrastructure) (14, 17-20). In an attempt to ensure sustainability of interventions, projects often incorporate a combination of these factors into their intervention package.

This study examines various components of program sustainability with a focus on the sustainability of family planning programming service delivery after donor funding ends.

Description of the NURHI intervention

The Urban Reproductive Health Initiative (URHI), funded by the Bill & Melinda Gates Foundation, was inaugurated in 2009, with the broad goal of addressing and meeting the FP needs of the urban poor in four countries: India, Kenya, Nigeria, and Senegal (21, 22). In Nigeria, the first phase of the Nigerian Urban Reproductive Health Initiative (NURHI) was implemented between 2009 and early-2015 in the Federal Capital Territory (FCT), Ibadan, Ilorin, Kaduna, Benin and Zaria. The overall goal of the NURHI was to promote innovative FP delivery approaches and to increase modern contraceptive use in the six cities, with a focus on the urban poor. In 2015, the second phase of NURHI commenced with operations in three states: Lagos, Kaduna and Oyo. NURHI-2 expanded its focus beyond the initial urban approach to include state-level interventions in both rural and urban areas. NURHI-2 incorporated approaches to support sustainability of project activities and ultimately FP uptake in its intervention sites.

The NURHI intervention utilized a three-tiered intervention model comprising advocacy, demand generation and service delivery to achieve its goals (23). The focus of this paper is on the service delivery components of the NURHI program. The NURHI service delivery approach included the following activities:

(i) Health system strengthening such as the NURHI 72-hour makeover, community outreach, integration of FP services within existing maternal, neonatal and child health (MNCH) and HIV services; improving the Commodities Logistics Management System (CLMS) and setting up a referral system.

For peer review only - http://bmjopen.bmj.com/site/about/guidelines.xhtml

(ii) Quality improvement: This was achieved through competency-based training, integrated supportive supervision and on-the-job training, site orientation for NURHI high volume sites and distance education.

(iii) Promotion of Public Private Partnerships (PPP) to improve referrals across sectors and increase access to FP services.

The Measurement, Learning & Evaluation (MLE) Project undertook the impact evaluation of the NURHI program; this included baseline, midterm, and endline household and facility-based surveys (24-26). Post immediate impact effects, it is important to assess the sustainability of the NURHI interventions when funding ends. This informs whether immediate gains are maintained and whether there are longer-term system-level and program-level benefits of the FP program. It is also important to highlight areas that might require urgent interventions to maintain the achievements.

The focus of this paper is to describe the extent to which NURHI interventions were sustained and factors influencing sustainability in a city where NURHI activities came to a close in 2015 (Ilorin) and a city where activities were modified and continued as part of NURHI-2 (Kaduna). Two measures of sustainability: (i) the extent to which the project activities continued in the target health facilities following closeout and (ii) perspectives of facility staff about the continuation of immediate effects of the intervention on the target population after project close out. Factors promoting sustainability of the intervention activities were also explored.

Research questions

(i) Were there any changes in facility-level intervention activities after the NURHI-1 project closed?

(ii) Were there changes in immediate outcomes - attitudes and demand for FP after the project closed?

Methods

The NURHI Sustainability Study had two components: a quantitative component (27, 28); and a qualitative component from which data for the current paper is obtained. The qualitative phase of the NURHI Sustainability Study was led by researchers at the Centre for Population and Reproductive Health, College of Medicine, University of Ibadan, Nigeria with technical support from the MLE project at the Carolina Population Center in Chapel Hill, North Carolina, USA. Information was obtained from participants in Ilorin, Kwara state and Kaduna city, Kaduna state.

For peer review only - http://bmjopen.bmj.com/site/about/guidelines.xhtml

For the qualitative study, we undertook key informant interviews (KII) with government stakeholders, in-depth interviews (IDI) with health facility staff, and focus group discussions with women from the study communities. This paper is based on the in-depth interview data from facility staff as the objective is to assess sustainability of facility-based interventions. In addition to the interviews, research staff also documented the availability and state of interventions implemented during the NURHI-1 program using a structured checklist. These data collection methods were selected to enable investigators to obtain in-depth information from a range of facility stakeholders about the NURHI-1 interventions and underlying factors which facilitated or undermined sustainability.

Study Participants

Selection of the facilities for the qualitative study was informed by data obtained from the quantitative phase. In total, NURHI worked in 21 facilities in Ilorin and 23 facilities in Kaduna under NURHI-1. Quantitative data collected from facilities in 2017 were analyzed to ensure that a mix of facilities categorized as high and low sustaining were selected. Factors taken into consideration included variables that would have been influenced by NURHI programming such as availability of IEC materials, training of staff within the last year, contraceptive method availability, existence of an outreach program, and existence of a quality assurance committee. Based on this process, in-depth interviews were undertaken between June and July, 2018 with facility staff (family planning service providers) in ten facilities in Ilorin (five that had positive indication of sustainability and five with worse indicators related to sustainability) and six in Kaduna (three with better and three with worse indicators). A total of 31 health facility staff (20 in Ilorin and 11 in Kaduna) were interviewed including the medical director or designate and a staff directly involved in providing FP services in each of the selected facilities. The study over sampled family planning providers from Ilorin in order to get a more nuanced perspective of the impact of NURHI-1's closure. Only staff who had been working in the facility prior to commencement of the NURHI-1 intervention were eligible to participate. The total number of participants interviewed was deemed adequate to meet the study objectives, however, there was provision to recruit additional interviewees until saturation was attained (29).

Study instruments

The in-depth-interview guide and health facility checklist were developed by a consultative process and all researchers agreed on the final version. The instruments were translated into Yoruba and Hausa, the predominant indigenous languages in the study cities, and back-translated to ensure the original meanings were retained (30). The instruments were pretested among a comparable group of respondents and revised as required subsequently. A three-day training was conducted for all research staff who conducted the

For peer review only - http://bmjopen.bmj.com/site/about/guidelines.xhtml

interviews. The training comprised didactic lectures, case studies, role plays as well as practical field visits and interviews followed by presentations and feedback. The training was interactive and provided data collectors and the study investigators the opportunity to edit the instruments where necessary and discuss city specific issues which could arise and how to resolve these. In addition, the instruments were further reviewed following the training to ensure that locally acceptable terms were incorporated as required. The interviews were conducted by a team comprising an interviewer and a notetaker and each interview lasted approximately 45 minutes to more than an hour. The interviews were digitally recorded and the notetaker documented notes as well as non-verbal cues.

For peer review only

Analysis

Data analysis was guided by a content analysis (31) and conducted by a team led by a qualitative researcher from MLE and an in-country researcher. Recorded interviews and discussions were translated and transcribed verbatim. Random checks of up to 10% of all transcripts against the recordings were conducted by the analysis team leads to ensure that the interviews were accurately transcribed. The transcripts were then uploaded into ATLAS.ti software (Scientific Software, Berlin; V.7) and coded.

Codes were developed using both deductive and inductive approaches and codebook development was informed by guidelines on "Codebook Development for Team-Based Qualitative Analysis" by McQueen et al (1998) (32). This improved inter-coder agreement and facilitated coding and data analysis by the MLE and in-country team. The data collection team leads and interviewers were also involved in the process of code development and provided additional clarification as required.

Coding of the transcripts was conducted by a coding team comprised of a team lead and six coders. When discrepancies were noted, the coding team discussed these and the code to be applied was agreed on. The team lead independently conducted spot checks on randomly selected transcripts during the coding process. Coding continued until appropriate codes were applied to all segments of the transcripts (29). For the current paper, the authors read the coded transcripts again and developed additional sub-codes where required. Codes were categorized into themes for each of the objectives and cross-case analysis performed using ATLAS.ti (version 7). (Additional details on data analysis in Appendix I).

Ethical approval for the study was obtained from the National Research Ethics Committee (protocol number: NHREC Protocol Number NHREC/01/01/2007-25/04/2017) and the Institutional Review Board at the University of North Carolina at Chapel Hill (study number: 17-1215). All participants provided verbal consent and they were assured that their responses were confidential and they could discontinue the

For peer review only - http://bmjopen.bmj.com/site/about/guidelines.xhtml

interview at any point if they desired to do this. In addition, the interviews were all conducted in private locations where participants were free to express their opinions.

Patient or Public Involvement

Research assistants were recruited from the general public and participated in the data collection. Patients were not involved in the study.

Results

Characteristics of respondents interviewed in Ilorin and Kaduna

Thirty-one participants (20 FP providers in Ilorin and 11 in Kaduna) provided consent and were interviewed. One participant who initially agreed to take part in the study in Kaduna later declined for personal reasons.

Respondents' views on the sustainability of NURHI-1 activities and the factors promoting sustainability are presented in the following sub-sections:

i. Sustainability of NURHI-1 activities in Ilorin and Kaduna
ii. Sustainability of immediate outcomes i.e. improved FP awareness, perceptions and demand in Ilorin and Kaduna
iii. Factors promoting sustainability of the NURHI-1 intervention activities and demand for FP

Sustainability of NURHI-1 activities in Ilorin and Kaduna

Health facility staff in Ilorin and Kaduna were interviewed for their views about sustainability of the different facility-level NURHI-1 activities including advocacy and community mobilization, service delivery, and interventions to ensure quality services provision. Respondents in both cities, revealed that activities were either ongoing or had been discontinued. For ongoing activities, a few were being conducted to a comparable degree as occurred during the NURHI-1 intervention period while others were "struggling", i.e. they were no longer being carried out to the same extent as occurred while NURHI-1 was ongoing. These views cut across all categories of the NURHI interventions and were expressed by more respondents in Ilorin than in Kaduna.

Advocacy and community mobilization activities in Ilorin and Kaduna

Generally, the interviewees reported that the community-level advocacy and community mobilization activities undertaken by members of the NURHI Advocacy Core Group (ACG) were no longer being implemented to the same extent as during NURHI-1. The decline in implementation of these activities was

For peer review only - http://bmjopen.bmj.com/site/about/guidelines.xhtml

predominately reported by participants in Ilorin, where NURHI-1 had closed out. Notably, some participants in Kaduna, (where activities under NURHI-2 were still ongoing) also mentioned a decline in community-level advocacy and mobilization which might reflect the focus under NURHI-2 on state-level activities and not just targeting of Kaduna city.

An FP provider in Ilorin described the state of things since the close-out of NURHI-1:

> *"…you know when NURHI was here, they helped us with many things. They had social mobilisers. These social mobilisers, they gave them token money for them to go out, to go inside the town, call people to come and do family planning. We had the ACG [Advocacy Core Group] - they gave us money to do everything; but now, [there is] nobody to finance us."*

Another FP provider in Ilorin remarked,

> *"We don't depend on …mobilization [for awareness generation] again; people are just coming in [for services]. Unlike before, when they [NURHI] were on ground, the mobilizers will go around the town, they invite people, they give them cards to come to this facility; but now that NURHI has left - nothing like that again."*

Although most of the FP providers interviewed in Kaduna mentioned that community mobilization and outreach were still ongoing, a few FP providers remarked that there had been a decline in these activities. For example, a FP provider in Kaduna explained, *"…the outreach; mostly it is done in the facility. Which is rather 'in-reach' to me, but then, when NURHI phase 1 was on, they [community mobilizers] do go into the community and they look for a strategic place, within the community and bring in people"*.

<u>Sustainability of service delivery interventions in Ilorin and Kaduna</u>

Responses on the sustainability of the integration of FP into existing maternal, neonatal and child health (MNCH) and HIV services were similar in both Ilorin and Kaduna. Respondents in both cities remarked that the integration had been sustained and enjoyed support from staff in these other units within the health facility. A respondent in Kaduna affirmed,

> *"We've integrated it [family planning] with the lab and [services for] people living with HIV. There is a training we undergo. That's why the PMTCT unit is just here and the FP [unit] is here. So that it will not be far, so any person coming for PMTCT, if she's a married woman, they will advise her to come to the FP unit, so she'll come for the FP and we will counsel her. And there are some women that come here purposely for FP, then we send them to the lab for HIV test"*.

For peer review only - http://bmjopen.bmj.com/site/about/guidelines.xhtml

Although FP had been well integrated into existing service units, stock-out of FP commodities and consumables was a challenge in the post-NURHI-1 period. This was reported more often among respondents in Ilorin than in Kaduna. A provider in Ilorin stated,

> "*Earlier this year, even at present, if I show you our cupboard, there is no Implanon because the one that we have has finished, so we don't have. And I requested, I made requisition from my supervisor. She told me that it's not available and the IUCD too is not available".*

In Kaduna, a FP provider corroborated the issue of stockout of commodities and efforts made to ameliorate this at the facility level which included reaching out to other facilities for commodities, *"The facility cannot do anything. We used to go to Kaduna south, sometimes their supply comes from the federal level so we can get what we want exactly. Sometimes when we request for hundred, we get fifty so that is why we get the stock out."*

We noted that although some respondents in both cities initially said they did not experience commodity stock-out, after probing for details of availability of the different FP commodities, it was discovered that virtually all government facilities, in both Ilorin and Kaduna, had experienced stock-out of at least one of the methods since NURHI-1 came to a close. Thus, providers had sometimes been constrained to offer clients an alternate method if the preferred method was unavailable. Excerpts from an interview with a FP provider in Ilorin illustrating this is outlined below:

> **Respondent:** Since I've been here, I have not experienced any stock out, since February. Except the month that ehnm … … … "Depo" injection [Depo Provera], that injectable was very scarce.
>
> **Interviewer:** … …, so during that period when the Depo wasn't available, what did you do for clients that came to seek that particular method?
>
> **Respondent:** so, hmm, we still advised them [to take] Noristerat … … … we told them that if "Depo" is available we will give them...
>
> - FP Provider, Ilorin

A provider in Kaduna also described the situation thus:

> *"The problem of stock-out we normally have is from [inadequate supply in] the whole state and [occurs] once in a while, but not always. There was a time Implanon was not available and they will say they have not collected delivery from the Federal [level]. So, it will be a general issue. ... And the microgynon, we used to have stock-out of microgynon, - sometimes, not always."*

For peer review only - http://bmjopen.bmj.com/site/about/guidelines.xhtml

FP providers in private and tertiary health facilities in both cities did not report having problems with stock-out of commodities as they obtained commodities from other sources and paid for these. A FP provider in a tertiary facility in Ilorin explained, *"We don't have stock out. We have all the methods on ground now. We don't have stock out and they [clients] can come for any method they like. In this facility, we offer all methods".*

A provider in a private facility in Kaduna remarked, *"... once it is end of the month, I prepare a report so that there wouldn't be any stock out".*

The problem of stock out of FP consumables was quite common both in Ilorin and Kaduna. Many of the respondents had experienced stock-out of consumables such as cotton wool, gloves, needles and syringes, and bleach. A FP provider in Kaduna specifically mentioned the challenges with availability of consumables experienced since NURHI-1 transitioned to NURHI-2.

> *"You know now, they don't concentrate more in the urban, they are now in the rural areas, so like now the consumables they used to give us has reduced. [It's] not like before, not like NURHI-1 and that is understood because they are facing [focusing on] the rural areas, the grassroots."*

Stock out of consumables was not a problem in private facilities in either of the two cities as these facilities billed patients for services including FP services. They could thus afford to procure consumables privately and not depend on government supply or supply by NGOs.

<u>Sustainability of quality FP service provision in Ilorin and Kaduna</u>

Interventions to ensure quality FP service provision such as competency-based training and on-the-job training and site orientation for NURHI high volume sites had been significantly affected by the NURHI-1 close out in both study cities. Facility staff in Ilorin reported that the NURHI-type of trainings had become non-existent. A provider in Ilorin stated,

> *"At this time I want to implore the NGOs on the training and retraining of staff on FP. There are many staff that have interest, when there is no training for them, there is nothing we can do. Training and retraining are very, very necessary."*

A similar observation was made by FP providers in Kaduna; *"NURHI has not been training our staff again, that's one of the changes."*

For peer review only - http://bmjopen.bmj.com/site/about/guidelines.xhtml

In addition to the fact that NURHI trainings had been discontinued, providers mentioned that a number of the facility staff who had been trained had either retired, were approaching retirement or had relocated to other health facilities or states where they would be better remunerated. This had depleted the pool of trained FP providers.

Another important NURHI-1 intervention undertaken to strengthen the health system was the 72-hour makeover which involved facility renovation and supply of equipment. The majority of the activities implemented under the 72-hour makeover renovations had been sustained in both cities. The equipment supplied were still largely available and functional. Examples of quotes from FP providers in Ilorin and Kaduna illustrating the current state of the renovations carried out are below:

> *"The equipment are still intact and all other things that they put in place like; the scale, equipment to check their BP." (FP provider, Ilorin)*

> *"We are maintaining what they gave us to work with; like the instruments, the chairs outside. If they break, we need to repair them to make everything go well." (FP provider, Kaduna)*

A provider in Kaduna mentioned that he spearheaded maintenance activities within his facility. He commented, *"As you can see, it's not long they repaired this place. I actually did the repairs. From my micro plan, I did the repairs."*

Neither respondents in Ilorin nor those in Kaduna had witnessed a major large-scale renovation in their facilities since the end of NURHI-1, although minor repairs had been carried out. A provider in Kaduna commented, *"we have not been seeing some things again from them [NURHI]. Like all these chairs are from NURHI and the tables and some of the things are from NURHI. In fact, like the chairs, the tables and all these things in the facility - in this unit are from NURHI, but now we no longer see anything again."*

As part of the study, interviewers observed the premises of the facilities for evidence of sustainability of the 72-hour health facility renovations. These findings largely corroborated the reports of the interviewees. A higher proportion of respondents in Kaduna than in Ilorin affirmed that their facilities had benefited from the NURHI 72-hour makeover, however, the make-over plaques were seen in a higher proportion of facilities in Ilorin than in Kaduna. The environments around all the health facilities surveyed in Ilorin and most of facilities in Kaduna were clean and well-kept at the time of the visit. Many facilities in Ilorin and Kaduna had spaces allocated to FP which were conducive and offered privacy for clients. All the facilities in Ilorin and in Kaduna had FP IEC materials displayed and NURHI-IEC materials were seen in all but one facility in Kaduna (Table 1).

For peer review only - http://bmjopen.bmj.com/site/about/guidelines.xhtml

Table 1: Sustainability of facility renovations as observed by interviewers

	**Ilorin**	**Kaduna**
	n = 10	n = 6
	n (%)	n (%)
NURHI-72 hour makeover conducted	6 (60%)	5 (83.3)
NURHI 72-hour makeover plaque sighted	4 (66.7)	3 (60.0)
Clean and well-kept FP environment	10(100.0)	5 (83.3)
Conducive space that affords auditory and visual privacy allocated to FP	8 (80.0)	3 (50.0)
FP IEC materials seen	10 (100.0)	6 (100.0)
FP IEC materials produced by NURHI seen	10 (100.0)	5 (83.3)
FP IEC materials targeting adolescents and youth seen	1 (10.0)	1(16.7)
FP IEC materials produced by NURHI targeting adolescents and youth seen	1 (100.0)	0 (0)

Overall, lack of funds was a major factor cited as responsible for the cessation or decline in activities in Ilorin and Kaduna. While respondents in Ilorin attributed this lack of funds to the close-out of the NURHI-1 intervention, respondents in Kaduna often attributed this to the modification of the focus of the NURHI-2 interventions which expanded to include rural communities beyond Kaduna city. Hence some respondents in Kaduna were no longer experiencing the effect of the NURHI-2 activities compared to the situation under NURHI-1.

Sustainability of immediate outcomes of NURHI-1 interventions in Ilorin and Kaduna

Respondents in both cities affirmed that the NURHI-1 intervention had improved FP awareness, perceptions and demand for commodities in their catchment areas. They further acknowledged that although NURHI activities had declined, the effects of these activities were still being felt. A FP provider in Ilorin remarked,

> *"Before, there was not much of awareness - before NURHI came. People didn't know much about family planning. Even people that knew, they just knew; maybe about the pills and injectables. But*

For peer review only - http://bmjopen.bmj.com/site/about/guidelines.xhtml

now that people are aware that there is long acting [contraceptive], they tend to go for the long acting rather than the short [acting] ones."

She further added, *"We have people that have busy schedules, like those people working in the bank, you don't see them before, but now you see them coming for those long acting [methods], they come for IUCD or Implanon." (FP provider, Ilorin)*

Another provider in Ilorin affirmed that demand for FP services had increased as a result of the NURHI-1 intervention.

"It has increased because there is more awareness now, people get to know more about family planning and during the antenatal clinic, they are getting information. Even some, before they deliver, you will see them coming for counseling that immediately they deliver or as soon as they deliver, they want to come for family planning. So they are more knowledgeable now and this makes them to be coming." (FP provider, Ilorin)

Similar views were expressed by FP providers in Kaduna, who admitted that demand for FP, especially LARCs, had increased, as a result of the NURHI community awareness outreaches. One of the FP providers interviewed had this to say:

"More people are asking for it, you know it's a long term method and people are thinking for them to go, comeback after 2 months, still go and come back after 3 months, they just feel the long acting method is the best. They tell you, 'I prefer the long acting method because once I just put it on, I'll just go on my own, I'll forget I'm putting it, no need of me telling myself I have to go back in 2 – 3 months, so it's preferable I go for the long term method'." (FP provider, Kaduna)

The NURHI "Get It Together" edutainment radio programme was also noted to have contributed to improved awareness about FP and subsequently demand for FP commodities. This finding was reported by respondents in both Ilorin and Kaduna although the radio programme had been discontinued in Ilorin following closeout of NURHI-1.

Factors promoting sustainability of NURHI-1 activities

Generally, many of the activities were no longer being implemented to the same extent as occurred during the NURIH-1 period in both Ilorin and Kaduna. Activities were however better sustained in Kaduna than in Ilorin. Respondents in both cities provided insights into factors that had promoted sustainability of some

For peer review only - http://bmjopen.bmj.com/site/about/guidelines.xhtml

activities following NURHI-1 close-out in Ilorin and transition to NURHI-2 in Kaduna. These are presented under the following key themes that emerged: project-related factors, factors within the health facility and factors within the larger community.

Project-related factors promoting sustainability of NURHI activities in Ilorin and Kaduna

A key project-related factor that promoted sustainability was the integration of FP awareness generation and service provision into existing activities and services within the health facilities. This was reported by providers in both Ilorin and Kaduna. In addition, the 72-hour makeover gave the facilities a face-lift and attracted clients to the facility. A respondent in Ilorin when asked about the immediate effect of the makeover remarked, "*people are trooping in; we have inflow, high inflow of ehn… client to this facility because through the light [sign] that was placed outside, it attracted people even those that are bringing their children for treatment here in the hospital.*" (FP provider, Ilorin).

A provider interviewed in Ilorin stated that staff in the health education unit in the facility had incorporated FP information into routine immunization and antenatal care clinics thereby facilitating continuation of FP awareness creation activities. She explained:

> *"We normally have our National Programme on Immunization (NPI) sessions - Mondays and Wednesdays; then they talk to them about family planning during that period - Mondays, Wednesdays and Fridays. Especially for mothers who have just given birth and came for their routine immunization for their children. So we talk to them about family planning in this facility."*

Similar accounts were shared by FP providers in Kaduna. In addition, some facilities were said to have involved Voluntary Community Mobilizers (VCM) and Traditional Birth Attendants (TBAs) in creating awareness of FP within the community.

> *"Yes, the VCM are playing their own role, the TBA's too are playing their own role in family planning by educating women out there at the community level. Then during routine immunization where we gather many of the women that bring their children for immunization, we use that opportunity too because at that time we can really get some of them. Then during antenatal again, we educate them about choosing a method of their own choice. So there is great improvement." (FP provider, Kaduna)*

Health facility-related factors promoting sustainability of NURHI activities in Ilorin and Kaduna

Some respondents cited motivation of health facility staff as a factor promoting sustainability of NURHI activities in both cities. Staff demonstrated their motivation in various ways for example, using personal

For peer review only - http://bmjopen.bmj.com/site/about/guidelines.xhtml

allowances to purchase minor equipment and stepping down the FP training they had received from NURHI to other staff. A FP provider in Kaduna mentioned that he and other colleagues sometimes utilized their transportation stipends to purchase minor equipment. He explained, *"I was able to get these [pointing to a gallipot and some forceps] - some gallipots, forceps, from what they give us (our transport stipends)."*

Another FP provider in Kaduna explained that on account of the increased demand for FP, management of the facility planned to increase the number of staff in the family planning unit and provide training for them to enable them to provide quality FP services to the increasing number of clients. He explained thus:

> *"Because of the increase in number of patients, we plan to put more staff in the unit and then to do step down training and then teach them the procedures so that they can cope with the challenge and increased numbers of patients."*

In contrast, direct efforts by the facility management to increase the staff strength was not mentioned by the respondents in Ilorin.

<u>Factors within the larger community</u>

Two main factors within the larger community - the presence of other NGOs and to some extent, government efforts were said to have contributed to sustainability of NURHI activities. Respondents in Ilorin and in Kaduna (in facilities that were not included in the NURHI-2 intervention or where providers stated that the impact of NURHI activities had waned under NURHI-2) mentioned that other NGOs working to improve FP in their states had promoted sustainability of NURHI interventions. Some of these NGOs included Pathfinder International, Society for Family Health (SFH) and Marie Stopes International. These NGOs provided funds, FP training, commodities and consumables, and they were also involved in awareness generation activities. A special government-initiated intervention - Saving One Million Lives Initiative - was said to have contributed towards sustainability of some NURHI activities in Ilorin and Kaduna.

Providers in Ilorin mentioned that outreach, community mobilization and awareness creation activities sponsored by other NGOs within the state had contributed towards sustainability of some of the activities NURHI had implemented and had also sustaining FP demand generation. A FP provider in Ilorin explained,

> **"***We used to organize outreaches, we send some people out to go and tell people, to go from house to-house ..., just to encourage them to come out for family planning. ... especially Marie Stopes - you know Marie Stopes has taken over after NURHI left."*

For peer review only - http://bmjopen.bmj.com/site/about/guidelines.xhtml

Another FP provider in Ilorin expressed concern about the initial decline in clientele experienced when NURHI-1 came to an end. She however mentioned that the number of clients had again increased when another NGO commenced a FP intervention project in the city. She explained thus,

"Although when they [NURHI] were here - the population of clients then was higher. The moment they [NURHI] left, things fell apart. But now, when another NGO - Marie Stopes came, then we are improving again. Because, before in this clinic we had 500, 600 clients per month, this declined to 100- plus, but now we have improved to 200, 300 [clients]."

Additional ways through which other NGOS had contributed to the continuation of some of the NURHI activities were through training of health care workers in the provision of FP services and provision of IEC materials. NGOs had also been instrumental towards mitigating stockout of commodities and consumables in Ilorin and in some facilities in Kaduna.

Interviewer: Marie Stopes that you mentioned, how did they contribute to family planning service delivery in this facility?

Respondent: Thank you. Through their trainings, then those that they trained, trained others too, then the knowledge people gained was a sort of contribution to family planning especially on these new methods.

FP Provider, Ilorin

In Ilorin, the College of Nursing and Mid-wifery was said to be organizing a training in FP use which all nurses working in FP units were encouraged to attend in order to acquire necessary skills. The training was however not free and would only hold if a minimum number of participants were registered. A FP provider provided the following information:

"So that one [training] is for [organized by] the school of -, College of Nursing and Midwifery. They are the ones organizing that family planning stuff. They should normally have 50 students at a time; so if they don't have this number, it may delay the training. To strengthen family planning, four people went last year but this year we are planning for them to go so that every staff will have knowledge that they need about family planning. So that's the plan we are making here."

A special government-initiated intervention - Saving One Million Lives Initiative - was said to have contributed towards sustainability of some NURHI activities in Ilorin and Kaduna. Responses on government efforts to sustain the NURHI activities and provide funding for FP commodities and consumables and for minor repairs within the health facilities differed. While some respondents

For peer review only - http://bmjopen.bmj.com/site/about/guidelines.xhtml

acknowledged government efforts, others were of the opinion that the government had not done enough but that NGOs were the main supporters of FP services. The differing opinions were expressed by respondents in both Ilorin and Kaduna. A provider in Kaduna commented: *"the state government is actually trying. They make sure that there is no stock out".*

Another FP provider in Kaduna however held a contrary view and when asked if the state government or other organization had assisted with minimizing stock-out in the last five years, he responded, *"not really, it's just partners like Marie Stopes in collaboration with the state, then this NURHI, Planned Parenthood Federation of Nigeria (PPFN)".*

Discussion

The paper describes the extent to which the activities of NURHI-1 have been sustained in Ilorin two years after close-out of the initiative and in Kaduna where the program continued but with a slightly different scope and focus. Our findings revealed that in Ilorin, integration of FP awareness creation activities and FP services into existing MCH services and the renovations and equipment purchased as part of the 72-hour clinic makeover had been sustained. Community outreach and availability of commodities and consumables were not sustained to the same degree as they had been during the NURHI-1 period. The NURHI-facilitated FP training had been discontinued. The situation in Kaduna which is still benefiting from NURHI-2 support, was slightly better than in Ilorin, although respondents noted that interventions under NURHI-2 were not as robust as they had been during the NURHI-1 implementation. The NURHI-1 mid-term and immediate closeout evaluations reported much higher degrees of sustainability compared to findings in the current study conducted about three years after NURHI-1 closeout (24, 25). Our findings thus indicate a progressive decline in project activities as the duration from NURHI-1 close-out to time of evaluation increases.

Common reasons given by respondents in both cities for failure to sustain NURHI-1 activities were inadequate funds and problems with the commodity supply chain, which could also be linked to funding. Other authors have underscored the importance of funding in sustaining health and other programme interventions (33-37). Shediac-Rizkallah and Bone (1998) emphasize the need for projects to have strategies to facilitate gradual financial self-sufficiency in order to promote sustainability (35). The transition from NURHI-1 to NURHI-2 in selected cities including Kaduna could be an attempt to facilitate this gradual financial self-sufficiency, however, our findings suggest that this transition might not achieve this effect as many NURHI-1 activities were declining in Kaduna as was also reported in Ilorin.

Stockout of FP commodities and consumables was reported in both study cities in spite of the robust advocacy component of NURHI-1 which contributed to the creation of government guidelines and

For peer review only - http://bmjopen.bmj.com/site/about/guidelines.xhtml

strategies such as the Nigeria Family Planning Blueprint (Scale-Up Plan) 2014 (38), a dedicated FP budget line, and increased fund allocation to FP by the Nigerian government. With these government interventions in place, it is expected that there would have been adequate funding to continue NURHI project activities. This was however not the case and a possible explanation might be that although these policies and guidelines had been approved, the systems to ensure prompt implementation at all levels, especially down to the health facility levels, were still nascent and yet to be fully operational.

Findings on sustainability of immediate outcomes of NURHI-1 suggested that FP awareness and demand remained high even after close-out in Ilorin and in the continuation phase in Kaduna. That said, concerns about declines in FP demand were raised by some respondents in Ilorin. This sustained FP awareness and demand were attributed to the widespread community advocacy and mobilization, awareness creation within the facilities as well as availability of a range of FP methods during Phase 1. Reviews of literature on programme sustainability have reported that community engagement which was an important aspect of NURHI-1 promotes programme sustainability by changing social norms in support of the programme. (18). It is important to note that the recurrent stock-out of commodities and consumables experienced in Ilorin and to some extent in Kaduna could potentially erode these immediate gains. It is thus important for the issue of stock-out to be urgently addressed and for measures to be put in place in the NURHI-2 sites to prevent re-occurrence of stock-out.

Factors which promoted sustainability from the respondents' views were integration of FP awareness generation and services into existing services and programmes, the 72-hour clinic makeover that increased patronage, and activities of other NGOs and some special government intervention programmes. NURHI-1 activities that leveraged on existing structures such as integration of FP service provision within existing Maternal, Neonatal and Child Health (MNCH) and HIV services were sustained. Integration of project activities within existing programs and services has been identified as an important factor that promotes sustainability (35). Integrating FP into other existing health services is one of the key strategies being explored to improve FP availability and demand in Nigeria and this could also have contributed to sustainability of this intervention beyond the NURHI-1 period (38). The 72-hour clinic makeover included provision of equipment which were still functional at the time of the study. Sustainability of this intervention in the short-term could be attributed to the fact that if properly utilized, the equipment would typically last for a while before needing to be replaced. The collaborative approach adopted for the 72-hour makeover involved prioritization and costing of items that required replacement or repair by facility staff and key community members and use of direct labour for repairs. Although not mentioned by the respondents in either of the two cities, involving the staff in the process of renovation could have contributed to sustainability by promoting ownership and careful use of the equipment (39).

For peer review only - http://bmjopen.bmj.com/site/about/guidelines.xhtml

Staff motivation also contributed to sustainability of some activities in both cities such as their efforts to stepdown the FP training received to newer staff. While the fidelity of this step-down training might not be the same as the initial training provided, using a training-of-trainers model and providing training manuals can enhance sustainability and scale up of training interventions (40, 41). In Ilorin, a respondent mentioned that the Nursing and Midwifery College had a FP training targeting the nurses. This suggests that embedding the training component within an existing system or collaboration with existing professional organizations could be useful in sustaining manpower capacity building efforts of health projects. This further emphasizes the importance of integrating project activities within existing systems and structures. The activities of other NGOs, which sustained some activities, cannot be said to promote sustainability in a real sense as the activities of the new NGO are also limited by the scope and time line of the project. Similarly government special programmes, if not integrated into existing programmes for continuity, are also limited in duration.

On the whole, challenges with sustainability of activities were reported more by respondents in Ilorin compared to their counterparts in Kaduna. This was probably because NURHI activities came to a close in 2015 in Ilorin (a NURHI-1 site), whereas activities were ongoing in a modified format in Kaduna as part of NURHI-2. A more gradual close-out of NGO-driven activities to allow the government to fully takeover the program, as suggested by a stakeholder in Kaduna, might have provided more time for the mechanisms for ensuring that the effects of the approved increment in government funding for FP are launched and felt at the facility level; this might have prevented the decline in activities observed in Ilorin.

Limitations

Our study had a few limitations. We acknowledge the limitations inherent in qualitative research methods and thus do not attempt to over-generalize our study findings. Furthermore, we did not define a hypothesis to test factors associated with sustainability. Our respondents in both cities however provided detailed responses describing their views about factors that promoted sustainability of the NURHI intervention in their locations. The study participants' views on sustainability could have been limited based on their job description and cadre within the health system. In addition, findings on the impact of the interventions on sustaining FP demand were based on the views of the providers which could have been over-estimated. We however noted that the providers remarks did not only attest to sustained FP demand as some of them also mentioned that demand had reduced following close-out and only increased after another NGO commenced FP interventions. To improve the quality of our data, we selected a range of participants who were knowledgeable about the study questions and we compared responses across respondents within each city.

For peer review only - http://bmjopen.bmj.com/site/about/guidelines.xhtml

We also included a structured observation to objectively assess sustainability of some of the NURHI-interventions. Participants from the NURHI-2 sites could have experienced some difficulty in differentiating between NURHI-1 and NURHI2 interventions. In order to minimize the likelihood of incorrect reporting, we clarified this difference to participants in the NURHI-2 site and emphasize that the questions were based on NURHI-1 interventions. We have however reported comprehensive and in-depth findings obtained through rigorous qualitative methods and these provide useful insight into the extent to which the NURHI-1 activities were sustained.

Trustworthiness of Data

The design, data collection and analysis were informed by guidelines for ensuring trustworthiness of qualitative data described by Guba (1981) (42) and Shenton (2004) (43). These guidelines mention four issues (credibility, transferability, dependability, and confirmability) as key to achieving trustworthiness of qualitative data. The investigators made efforts to ensure the study design, data collection and analysis met these criteria (please see details in Appendix II).

Conclusion

On the whole, results of this sustainability study revealed that while a few of the NURHI initiated interventions were sustained, many more had declined or discontinued, particularly in Ilorin where the program ended. In addition, although most activities were not being carried out as before, their effects such as improved demand for family planning commodities and improved perception about family planning were reportedly still being felt. These findings affirm the importance of incorporating a range of strategies to promote sustainability of donor projects that aim to improve family planning demand and uptake in our setting. Lack of funding for continuing project activities was a major deterrent to sustainability reported by our respondents, hence it is important for donor projects to develop a plan which facilitates gradual financial self-sufficiency of the project beneficiary so that activities can be sustained following project close-out. Selecting project activities which align with government plans and integrating these activities within existing services could also increase the likelihood of sustainability. Finally utilizing a training model that not only improves the competence of the trainees but also builds their capacity to step down the training could be useful for promoting sustainability of similar donor-funded projects.

Funding statement

This study was funded by a grant from the Bill & Melinda Gates Foundation to the Carolina Population Center at the University of North Carolina at Chapel Hill. This research also received support from the Population Research Infrastructure Program (P2C HD050924) awarded to the Carolina Population Center

For peer review only - http://bmjopen.bmj.com/site/about/guidelines.xhtml

at The University of North Carolina at Chapel Hill by the Eunice Kennedy Shriver National Institute of Child Health and Human Development. The contents of this article are solely the responsibility of the authors and do not necessarily represent the official views of CPC or the Bill & Melinda Gates Foundation.

Competing interests statement

Authors have no known potential conflict of interest. An abstract based on our findings has been presented at the International Family Planning Conference in Rwanda

Authors' contribution

AO was the in-country study supervisor for the study and contributed to the review of the protocol, editing and revision of the study instruments, training and supervision of field staff, data collection and analysis.

CM was the qualitative project investigator and involved in development of the protocol and study instruments, training and supervision of field staff and data analysis.

LC was a project investigator and involved in development of the protocol and study instruments and data analysis

IS was the study Principal Investigator and provided oversight for the entire project. She was also involved in development of the protocol and study instruments and review of the data.

TB was the in-country field supervisor and contributed to the review of the protocol, editing and revision of the study instruments, training and supervision of field staff, data collection and review of the data.

OO was the in-country principal investigator and provided in-country oversight for the project. He contributed to the review of the protocol, editing and revision of the study instruments, training of field staff supervision of data collection and review of the data

All authors contributed to the write-up and revision of the manuscript and approved the final version submitted for publication

Acknowledgements

We acknowledge all study participants and the research staff who worked on the project

Data Availability

The dataset associated with the paper are available on request from the Project Principal Investigator.

References

1. Ahmed S, Li Q, Liu L, Tsui AO. Maternal deaths averted by contraceptive use: an analysis of 172 countries. The Lancet. 2012;380(9837):111-25.

For peer review only - http://bmjopen.bmj.com/site/about/guidelines.xhtml

2. Canning D, Schultz TP. The economic consequences of reproductive health and family planning. The Lancet. 2012;380(9837):165-71.
3. Cates W, Karim QA, El-Sadr W, Haffner DW, Kalema-Zikusoka G, Rogo K, et al. Family planning and the millennium development goals. Science. 2010;329(5999):1603-.
4. Cleland J, Bernstein S, Ezeh A, Faundes A, Glasier A, Innis J. Family planning: the unfinished agenda. The Lancet. 2006 2006/11/18/;368(9549):1810-27.
5. Cleland J, Conde-Agudelo A, Peterson H, Ross J, Tsui A. Contraception and health. The Lancet. 2012;380(9837):149-56.
6. Starbird E, Norton M, Marcus R. Investing in family planning: key to achieving the sustainable development goals. Global Health: Science and Practice. 2016;4(2):191-210.
7. Creanga AA, Gillespie D, Karklins S, Tsui AO. Low use of contraception among poor women in Africa: an equity issue. Bulletin of the World Health Organization. 2011;89:258-66.
8. Cleland JG, Ndugwa RP, Zulu EM. Family planning in sub-Saharan Africa: progress or stagnation? Bulletin of the World Health Organization. 2011;89:137-43.
9. Tsui AO, Brown W, Li Q. Contraceptive Practice in Sub-Saharan Africa. Population and development review. 2017;43(Suppl Suppl 1):166-91. PubMed PMID: 29081552. Epub 03/31.
10. National Population Commission (NPC) [Nigeria] and ICF International. Nigeria Demographic and Health Survey 2013. Abuja, Nigeria and Rockville, Maryland, USA: NPC and ICF International, 2014.
11. Mwaikambo L, Speizer IS, Schurmann A, Morgan G, Fikree F. What Works in Family Planning Interventions: A Systematic Review. Studies in Family Planning. 2011;42(2):67-82.
12. Bamberger M, Cheema S. Case studies of project sustainability: implications for policy and operations from Asian experience: The World Bank; 1990.
13. Abrams L, Palmer I, Hart T. Sustainability management guidelines. Pretoria: Department of Water Affairs and Forestry. 1998.
14. Shediac-Rizkallah MC, Bone LR. Planning for the sustainability of community-based health programs: conceptual frameworks and future directions for research, practice and policy. Health education research. 1998;13(1):87-108.
15. Weiss H, Coffman J, Bohan-Baker M, editors. Evaluation's role in supporting initiative sustainability. Proceedings of the Fifth Bi-Annual Meeting of the Urban Seminar Series on Children's Health and Safety at Harvard University, Cambridge, MA, USA; 2002.
16. Shelton RC, Cooper BR, Stirman SW. The sustainability of evidence-based interventions and practices in public health and health care. Annual review of public health. 2018;39:55-76.
17. Riggs K. Strategies for Sustainability of Grant-funded Programs. 2012.
18. Iwelunmor J, Blackstone S, Veira D, Nwaozuru U, Airhihenbuwa C, Munodawafa D, et al. Toward the sustainability of health interventions implemented in sub-Saharan Africa: a systematic review and conceptual framework. Implementation Science. 2016 March 23;11(1):43.
19. Banach M, Gregory PJ. Essential tasks, skills, and decisions for developing sustainable community-based programs for children, youth, and families at risk. Journal of Extension. 2001;39(5):n5.
20. Chambers DA, Glasgow RE, Stange KC. The dynamic sustainability framework: addressing the paradox of sustainment amid ongoing change. Implementation Science. 2013 October 02;8(1):117.
21. NURHI Nigerian Urban Reproductive Health Initiative. NURHI Overview 2013 [cited 2019 4 February, 2019].
22. Guilkey D, Speizer I, Lance P. Study Design for the Measurement, Learning & Evaluation Project. Project for the Urban Reproductive Health Initiative, ed. 2009.
23. Krenn S, Cobb L, Babalola S, Odeku M, Kusemiju B. Using behavior change communication to lead a comprehensive family planning program: the Nigerian Urban Reproductive Health Initiative. Global health, science and practice. 2014;2(4):427-43. PubMed PMID: 25611477. eng.

For peer review only - http://bmjopen.bmj.com/site/about/guidelines.xhtml

24. Measurement Learning Evaluation Project Nigeria Team. Evaluation of the Nigerian Urban Reproductive Health Initiative (NURHI) Program. Studies in family planning. 2017;48(3):253-68.
25. Measurement Learning and Evaluation (MLE) Project NPCN, Data Research and Mapping Consult Ltd,. Measurement, Learning & Evaluation of the Urban Reproductive Health

Initiative: Nigeria, 2012 Midterm Survey [TWP 2-2013]. Chapel Hill, NC, USA, 2013.
26. Measurement Learning and Evaluation (MLE) Project NPCN, Data Research and Mapping Consult, Ltd,. Measurement Learning and Evaluation of the Urban Reproductive Health Initiative: Nigeria 2015 Endline Survey. [TWP 2-2015]. Chapel Hill, NC, USA: Measurement, Learning and Evaluation Project; 2015, 2015.
27. Measurement Learning & Evaluation (MLE) Project Team. Assessing Sustainability of NURHI Programming: Results from Facility-Level Survey. Chapel Hill (NC): MLE Project: November, 2018.
28. Measurement LEMPT. Assessing Sustainability of NURHI Programming: Results from Household-Level Survey. Chapel Hill (NC): MLE Project: November, 2018.
29. Hennink M, Hutter I, Bailey A. Qualitative Research Methods. London: SAGE Publications Ltd; 2011. 304 p.
30. Brislin RW. The wording and translation of research instruments. 1986.
31. Flick U. The SAGE handbook of qualitative data analysis: Sage; 2013.
32. MacQueen KM, McLellan E, Kay K, Milstein B. Codebook Development for Team-Based Qualitative Analysis. CAM Journal. 1998;10(2):31-6.
33. Iwelunmor J, Newsome V, Airhihenbuwa CO. Framing the impact of culture on health: a systematic review of the PEN-3 cultural model and its application in public health research and interventions. Ethnicity Health. 2014;19.
34. Scheirer MA. Is sustainability possible? A review and commentary on empirical studies of program sustainability. American Journal of Evaluation. 2005;26(3):320-47.
35. Shediac-Rizkallah MC, Bone LR. Planning for the sustainability of community-based health programs: conceptual frameworks and future directions for research, practice and policy. Health Educ Res. 1998;13(1):87-108.
36. U.S. Department of Health and Human Services Office of Adolescent Health. Building Sustainable Programs: The Framework. United States: 2014.
37. Savaya R, Spiro SE. Predictors of sustainability of social programs. American Journal of Evaluation. 2012;33(1):26-43.
38. Federal Government of Nigeria. Nigeria Family Planning Blueprint (Scale-Up Plan) October 2014. Abuja, Nigeria2014.
39. Nigerian Urban Reproductive Health Initiative (NURHI). 72 Hour Clinic Makeover 2013 [cited 2020 5 September,]. Available from: https://www.nurhitoolkit.org/program-areas/service-delivery/72-hour-clinic-make-over#.X1OOvHlKjIU.
40. Ray ML, Wilson MM, Wandersman A, Meyers DC, Katz J. Using a training-of-trainers approach and proactive technical assistance to bring evidence based programs to scale: An operationalization of the interactive systems framework's support system. American journal of community psychology. 2012;50(3-4):415-27.
41. Orfaly RA, Frances JC, Campbell P, Whittemore B, Joly B, Koh H. Train-the-trainer as an educational model in public health preparedness. Journal of Public Health Management and Practice. 2005;11(6):S123-S7.
42. Guba EG. Criteria for assessing the trustworthiness of naturalistic inquiries. Educational Technology Research and Development. 1981;29(2):75-91.
43. Shenton AK. Strategies for ensuring trustworthiness in qualitative research projects. Education for Information 2004;22:63–75.

For peer review only - http://bmjopen.bmj.com/site/about/guidelines.xhtml

For peer review only

For peer review only - http://bmjopen.bmj.com/site/about/guidelines.xhtml

Appendix 1

Data Analysis for the NURHI Sustainability study

Codes were developed using both deductive and inductive approaches and codebook development was informed by guidelines on "Codebook Development for Team-Based Qualitative Analysis" by McQueen et al (1998) (33). This improved inter-coder agreement and facilitated coding and data analysis by the MLE and in-country team. Code development commenced as soon as the first set of transcripts were available. The qualitative team leads read through each transcript and developed an initial list of codes and code definitions to discuss with the larger coding team. The codes were then applied to a set of transcripts and additional codes and sub-codes were developed, and the list of codes refined as needed. This process was repeated until a final list of codes and code definitions was agreed on. Examples of quotes relating to each code were provided and reviewed following which the final codebook was agreed upon and shared with the coding team. The data collection team leads and interviewers were also involved in the process of code development and provided additional clarification as required.

Coding of the transcripts was conducted by a coding team comprised of a team lead and six coders. Two coders first independently coded one transcript and this was presented and reviewed by the coding team. Discrepancies in coding were extensively discussed and the codes to be used in these instances agreed on by the team. The remaining transcripts were then assigned to members of the coding team. Each transcript was initially coded by one person following which another coder independently reviewed the coded transcript. When discrepancies were noted, the coding team discussed these and the code to be applied was agreed on. The team lead independently conducted spot checks on randomly selected transcripts during the coding process. Coding continued until appropriate codes were applied to all segments of the transcripts (29). For the current paper, the authors read the coded transcripts again and developed additional sub-codes where required. Codes were categorized into themes for each of the objectives and cross-case analysis performed using ATLAS.ti (version 7).

For peer review only

Appendix II

Trustworthiness of the qualitative data

To ensure credibility of our data, the qualitative study was led by qualitative experts from Nigeria and a range of data collection methods were employed to obtain comprehensive and accurate information. We selected a range of respondents in order to obtain rich and in-depth information and facilitate triangulation of responses across the respondents and the questions in the data collection guides were framed to ensure detailed information was provided. The initial findings were also discussed with members of the core team and other researchers who were knowledgeable in the FP field and this provided opportunity for peer scrutiny. During the analysis phase, the investigators referred to the field notes to obtain additional information and clarifications, conducted member checks as necessary and compared their findings to other related studies. To improve transferability of methods, the results were interpreted within the context of the study sites. Our use of a protocol that all investigators made input into promoted dependability of our findings. Furthermore, the data collection process was conducted in line with the protocol and this process can be replicated. To ensure confirmability of our findings, the investigators conducted member checks, regularly discussed the findings with one another and with the data collection staff to ensure that the ideas of the respondents were retained, and investigators did not interpret the findings based on their own pre-formed ideas. Our findings are thus comprehensive and contribute significantly to the existing body of knowledge on sustainability of donor-funded projects

For peer review only

Table 1 Consolidated criteria for reporting qualitative studies (COREQ): 32-item checklist

Domain 1: Research team and reflexivity		Page numbers
Personal Characteristics		
1. Interviewer/facilitator Which author/s conducted the interview or focus group?	None of the authors conducted the interviews. Authors conducted the qualitative training and trained field assistants with experience in moderating and conducting in depth interviews and focus group discussions conducted the interviews	Page 8<br><br>“”The interviews were conducted by a team comprising an interviewer and a notetaker”<br><br>Penultimate sentence under the sub-heading, “study instruments”
2. Credentials What were the researcher’s credentials? E.g. PhD, MD	Researchers’ credentials have been entered in the author section of the journal. All researchers have a postgraduate degree including Masters, PhD, Medical fellowship	These were filled out in the online form
3. Occupation What was their occupation at the time of the study?	Researchers and University faculty, lecturers and honorary hospital consultants, consultants, data officers	This information is not in the manuscript
4. Gender Was the researcher male or female?	The team comprised male and female researchers	Information is not in the manuscript
5. Experience and training What experience or training did the researcher have?	All researchers have training and experience in research conceptualization, design, implementation, monitoring and evaluation. The qualitative aspect of the study was led by researchers with experience in qualitative research methods.	This is not in the manuscript
Relationship with participants		None
6. Relationship established Was a relationship established prior to study commencement?	A professional relationship was established. The interviewees worked in health facilities under the NURHI programme and NURHI staff at the headquarters assisted in identifying the facilities. The in-country research team also paid advocacy visits to relevant officers within the study states and sites prior to commencement of data collection to ensure community	Information is not in the paper

For peer review only

	buy-in and willingness to participate in the study.	
7. Participant knowledge of the interviewer		
What did the participants know about the researcher? e.g. personal goals, reasons for doing the research	The reason for the study was explained to all participants when they were contacted. This was further explained to then when they were taken through the informed consent process. Personal information about the researchers was not shared with the interviewees	Page 9. First paragraph on ethics
8. Interviewer characteristics What characteristics were reported about the interviewer/facilitator? e.g. Bias, assumptions, reasons and interests in the research topic	Nil. Trained research assistants conducted the interviews	
Domain 2: study design		
Theoretical framework		
9. Methodological orientation and Theory		
What methodological orientation was stated to underpin the study? e.g. grounded theory, discourse analysis, ethnography, phenomenology, content analysis	Analysis was conducted using constant comparison	Pages 4 and 8
Participant selection		
10. Sampling How were participants selected? e.g. purposive, convenience, consecutive, snowball	Purposive sampling was undertaken. This was the preferred sampling technique as interviewees were expected to have been aware of the programme being evaluated and also meet certain criteria necessary for them to provide informed responses for the evaluation	Methods Page 7 under study participants
11. Method of approach How were participants approached? e.g. face-to-face, telephone, mail, email	They were approached face-to-face	Methods Page 7 under study participants
12. Sample size How many participants were in the study?	Data on 31 participants were analyzed for the current study	Methods Page 7 under study participants
13. Non-participation How many people refused to participate or dropped out? Reasons?	Only one participant dropped out. He had a busy schedule and could not continue with the interview as his attention was requiree	Methods Page 9 under participant characteristics

For peer review only

Setting		
14. Setting of data collection Where was the data collected? e.g. home, clinic, workplace	Data was collected at a venue convenient for the participant. This was the office in all cases	Methods<br>Under study participants, page 7
15. Presence of non-participants Was anyone else present besides the participants and researchers?	No. For some of the senior cadre staff interviewed, other staff occasionally came in to their office to see them, however these interruptions were minimal and confidentiality of responses was ensured.	
16. Description of sample What are the important characteristics of the sample? e.g. demographic data, date	Some non-identifying demographic characteristics – gender, position/ job description, state where data was collected.	Methods<br>Their job description is mentioned<br>under study participants, page 7
Data collection		
17. Interview guide Were questions, prompts, guides provided by the authors? Was it pilot tested?	An interview guide was utilized. The guides were pretested prior to the interviews	Methods-Study instruments, page 7
18. Repeat interviews Were repeat interviews carried out? If yes, how many?	No	
19. Audio/visual recording Did the research use audio or visual recording to collect the data?	Research used audio recording	Methods – Page 8. Last sentence under study instruments
20. Field notes Were field notes made during and/or after the interview or focus group?	Field notes were made during the interview and expanded after the interview	Methods – Page 8. Last sentence under study instruments
21. Duration What was the duration of the interviews or focus group?	Approximately an hour – about 45 minutes to more than an hour (an hour thirty minutes	Methods – Page 8. Last sentence under study instruments
22. Data saturation Was data saturation discussed?	Yes. This was discussed	Methods-Study instruments, page 7
23. Transcripts returned Were transcripts returned to participants for comment and/or correction?	Transcripts were not returned to participants to correct. Participants were called to clarify issues during the analysis phase and given a brief summary of their responses	Trustworthiness<br>Page 22
Domain 3: analysis and findings		
Data analysis		

For peer review only

24. Number of data coders How many data coders coded the data?	At least two coders coded each transcript. There were a total of six coders for the entire study. The data presented in the current paper is based on KII findings only. The process of code development was informed by guidelines I the book by McQueen et al (1998) "Codebook Development for Team-Based Qualitative Analysis"	Methods –Analysis: page 8
25. Description of the coding tree Did authors provide a description of the coding tree?	Not in the publication	
26. Derivation of themes Were themes identified in advance or derived from the data?	Themes were derived in advance and also from the data (both deductive and inductive theme derivation was conducted)	Methods<br>- Data analysis, pages 8 and 9
27. Software What software, if applicable, was used to manage the data?	ATLAS.ti software (Scientific Software, Berlin; V.7)	Methods<br>Data analysis, page 9
28. Participant checking Did participants provide feedback on the findings?	Yes. Some participants were called to clarify responses and interpretations given to responses during the analysis process	Trustworthiness<br>Page 22
Reporting		
29. Quotations presented Were participant quotations presented to illustrate the themes / findings? Was each quotation identified? e.g. participant number	Yes	Results Pages 9 to 20
30. Data and findings consistent Was there consistency between the data presented and the findings?	Yes	Results Pages 9 to 20
31. Clarity of major themes Were major themes clearly presented in the findings?	Yes	Results Pages 9 to 20
32. Clarity of minor themes Is there a description of diverse cases or discussion of minor themes?	Yes	Results Pages 9 to 20

For peer review only
